# A carcinogenic trigger to study the function of tumor suppressor genes in *Schmidtea mediterranea*

**DOI:** 10.1242/dmm.032573

**Published:** 2018-08-16

**Authors:** Andromeda Van Roten, Amal Zohir Abo-Zeid Barakat, Annelies Wouters, Thao Anh Tran, Stijn Mouton, Jean-Paul Noben, Luca Gentile, Karen Smeets

**Affiliations:** 1 Zoology: Biodiversity and Toxicology, Hasselt University–Campus Diepenbeek, Agoralaan 1, Gebouw D, 3590, Diepenbeek, Belgium; 2 Planarian Stem Cell Laboratory, Max Planck Institute for Molecular Biomedicine, von Esmarch-str. 54, 48149, Münster, Germany; 3 Zoology: Biodiversity and Toxicology, Hasselt University–Campus Diepenbeek, Agoralaan 1, Gebouw D, 3590 Diepenbeek, Belgium; 4 Pluripotency and Regeneration Group, Fraunhofer Institute for Biomedical Engineering, Joseph-von-Fraunhofer-Weg 1, 66280, Sulzbach, Germany; 5 European Research Institute for the Biology of Ageing, University Medical Center Groningen, University of Groningen, 9713, Groningen, The Netherlands; 6 Biomedical Research Institute, Hasselt University and Transnationale Universiteit Limburg, School of Life Sciences, 3590, Diepenbeek, Belgium; 7 Planarian Stem Cell Laboratory, Max Planck Institute for Molecular Biomedicine, von Esmarch-str. 54, 48149, Münster, Germany; 8 Zoology: Biodiversity and Toxicology, Hasselt University–Campus Diepenbeek, Agoralaan 1, Gebouw D, 3590, Diepenbeek, Belgium

**Keywords:** Planarian, Cadmium, Carcinogens, Matrix-metalloproteinases, Stem cells, Tumor suppressor genes

## Abstract

Planarians have been long known for their regenerative ability, which hinges on pluripotency. Recently, however, the planarian model has been successfully established for routine toxicological screens aimed to assess overproliferation, mutagenicity and tumorigenesis. In this study, we focused on planarian tumor suppressor genes (TSGs) and their role during chemically induced carcinogenic stress in *Schmidtea mediterranea*. Combining *in silico* and proteomic screens with exposure to human carcinogen type 1A agent cadmium (Cd), we showed that many TSGs have a function in stem cells and that, in general, exposure to Cd accelerated the onset and increased the severity of the observed phenotype. This suggested that the interaction between environmental and genetic factors plays an important role in tumor development in *S. mediterranea*. Therefore, we further focused on the synergistic effects of Cd exposure and *p53* knockdown (KD) at the cellular and molecular levels. Cd also produced a specific proteomic landscape in homeostatic animals, with 172 proteins differentially expressed, 43 of which were downregulated. Several of these proteins have tumor suppressor function in human and other animals, namely Wilms Tumor 1 Associated Protein (WT1), Heat Shock Protein 90 (HSP90), Glioma Pathogenesis-Related Protein 1 (GLIPR1) and Matrix Metalloproteinase B (Smed-MMPB). Both *Glipr1* and *MmpB* KD produced large outgrowths, epidermal lesions and epidermal blisters. The epidermal blisters that formed as a consequence of *Smed-MmpB* KD were populated by smedwi1^+^ cells, many of which were actively proliferating, while large outgrowths contained ectopically differentiated structures, such as photoreceptors, nervous tissue and a small pharynx. In conclusion, *Smed-MmpB* is a planarian TSG that prevents stem cell proliferation and differentiation outside the proper *milieu*.

## Abstract (precis)

**Summary:** Combining exposure to the human carcinogen cadmium with *in silico* and proteomic top-down approaches, we disclosed the conserved function of two novel tumor suppressor genes in planarian.

## INTRODUCTION

Regeneration is the process by which a complex sequence of cellular and molecular events coordinates the rebuilding of lost or damaged body parts, restoring their structural and functional integrity (Charni et al., 2017). Stem cells are fundamental players of regeneration, as they proliferate and differentiate to regrow the missing body parts (Beausejour and Campisi, 2006; Gentry and Jackson, 2013). However, highly proliferative cells are prone to replication errors, which makes them more sensitive to environmental agents (Preston-Martin et al., 1990; Tomasetti and Vogelstein, 2015, 2017). An in-depth understanding of the mechanisms underlying regeneration and tissue repair is therefore fundamental for developing regenerative therapies that effectively prevent tumorigenesis.

Hyperproliferation is controlled by tumor suppression mechanisms. These mechanisms rely on tumor suppressor genes (TSGs), which prevent uncontrolled proliferation by either repressing cell cycle progression or triggering apoptosis (Beausejour and Campisi, 2006). Different types of TSGs exist. Gatekeeper TSGs (e.g. *WT1* and *APC*) regulate cell proliferation; caretaker TSGs (e.g. *BRCA1*, *MSH2* and *MLH1*) maintain genome stability by modulating DNA repair. Mutations of these genes lead to genomic instability and cancer (Deng and Scott, 2000; Kwong and Dove, 2009; Levitt and Hickson, 2002; Scholz and Kirschner, 2011). Landscaper TSGs act on the cell niche, by regulating epithelial–epithelial and epithelial–stromal interactions. Their mutations promote cell transformation, such as the epithelial–mesenchymal transition, which often results in tissue invasion (Bullions et al., 1997; Hanahan and Weinberg, 2011). Some TSGs play more than one role, as does *P53* (also known as *TP53*), which has both gatekeeper and caretaker functions (Lane, 1992) and during regeneration preserves the integrity of the genome (Charni et al., 2017). Planarian *Schmidtea mediterranea*, a true master of regeneration (Gentile et al., 2011; Reddien and Sanchez Alvarado, 2004), has a single *p53* gene, which is expressed in stem cells and post-mitotic progeny. It regulates the homeostasis of the stem cell compartment and, in its absence, planarians develop outgrowths (Pearson and Sanchez Alvarado, 2010), as they do following the knockdown (KD) of another TSG, *Smed-Pten* (Oviedo et al., 2008).

Genetic factors often act synergistically with environmental factors to promote tumorigenesis. Cadmium (Cd) is a relevant environmental contaminant, classified as human carcinogen type 1A (Akesson et al., 2008; IARC, 1993). Human exposure to Cd can cause different types of cancer (McElroy et al., 2006; Waalkes, 2003). The mechanisms through which Cd promotes tumorigenicity include inhibition of DNA repair, induction of oxidative stress, overexpression of proto-oncogenes and resistance to apoptosis (Achanzar et al., 2002; Hart et al., 2001; Jin and Ringertz, 1990; Joseph, 2009; Nair et al., 2015). In planarians, the effects of Cd exposure differ from species to species. Cd-induced tumorigenesis was never clearly observed in *S.* *mediterranea* (Plusquin et al., 2012). It was suggested that the stem cell system in *S. mediterranea* is able to evade carcinogenic initiation and/or progression, and that the observed Cd-induced proliferation burst acts as a controlled repair mechanism, rather than as an uncontrolled onset of carcinogenesis. In contrast, in *Dugesia dorotocephala*, Cd gives rise to benign tumors (Hall et al., 1986). A recent study suggested that, in *Dugesia tigrina*, Cd might exert its tumorigenic effects by inducing metalloproteinase-dependent stem cell overproliferation (Voura et al., 2017). Matrix metalloproteinases (MMPs) are a multigene family of enzymes involved in the proteolytic degradation and the remodeling of the extracellular matrix (ECM) (Kim et al., 2011). Many MMPs are involved in tumor development, progression and metastasis (Merdad et al., 2014; Yamada et al., 2010); others, like MMP8 and MMP19, were found to limit tumorigenesis and/or metastasis (Impola et al., 2005; López-Otín et al., 2009). Metalloproteinases are also involved in the process of regeneration. Four MMP genes were described in both *S. mediterranea* and *Dugesia* *japonica*: *Mmp1*, *Mmp2*, *MmpA* and *MmpB*. Downregulation of either *Mmp1* or *MmpA*, but not of *Mmp2* and *MmpB*, resulted in general tissue dysplasia, formation of lesions and impairment of regeneration (Isolani et al., 2013).

Genes that make up for or influence stem and tumor cell niches are potential therapeutic targets. With this study, we aimed to identify genes with tumor suppression function in the stem cell model organism *S. mediterranea*. Therefore, we functionally tested 20 planarian TSGs, two of which were identified in a proteomic screen as downregulated after exposure to Cd.

## RESULTS

In order to fully understand how regenerative tissues can maintain controlled growth, we challenged the regenerative planarian *S.* *mediterranea* with external carcinogenic exposure. We aimed to achieve a comprehensive overview of *S. mediterranea* TSGs and their role during carcinogenic stress, for which we used two independent approaches. In the *in silico* approach, SmedGD was searched for bona fide homologs of human TSGs. In the proteomics approach, both homeostatic and regenerating animals exposed to the human carcinogen Cd were compared. Candidate TSGs were functionally validated by double-stranded RNA (dsRNA)-mediated RNA interference (RNAi) in the presence of Cd. The thus far known planarian TSGs – *Smed-PTEN* (Oviedo et al., 2008), *Smed-p53* (Pearson and Sanchez Alvarado, 2010), *Smed-Chd4* (Scimone et al., 2010), *Smed-Rb* (Zhu and Pearson, 2013) and *Smed-Smg1* (González-Estévez et al., 2012) – were also included, as their function was not studied in the presence of carcinogenic compounds. Previously, Hollenbach and colleagues studied the role played by *Smed-Msh2* in neoblast survival under genotoxic stress caused by the DNA-alkylating compound N-methyl-N′-nitro-N-nitrosoguanidine (MNNG) (Hollenbach et al., 2011). *Smed-Msh2* and Smed-*Mlh1* were also investigated under Cd stress in the current study.

### TSG homologs in *S. mediterranea*

**Fig. 1.**
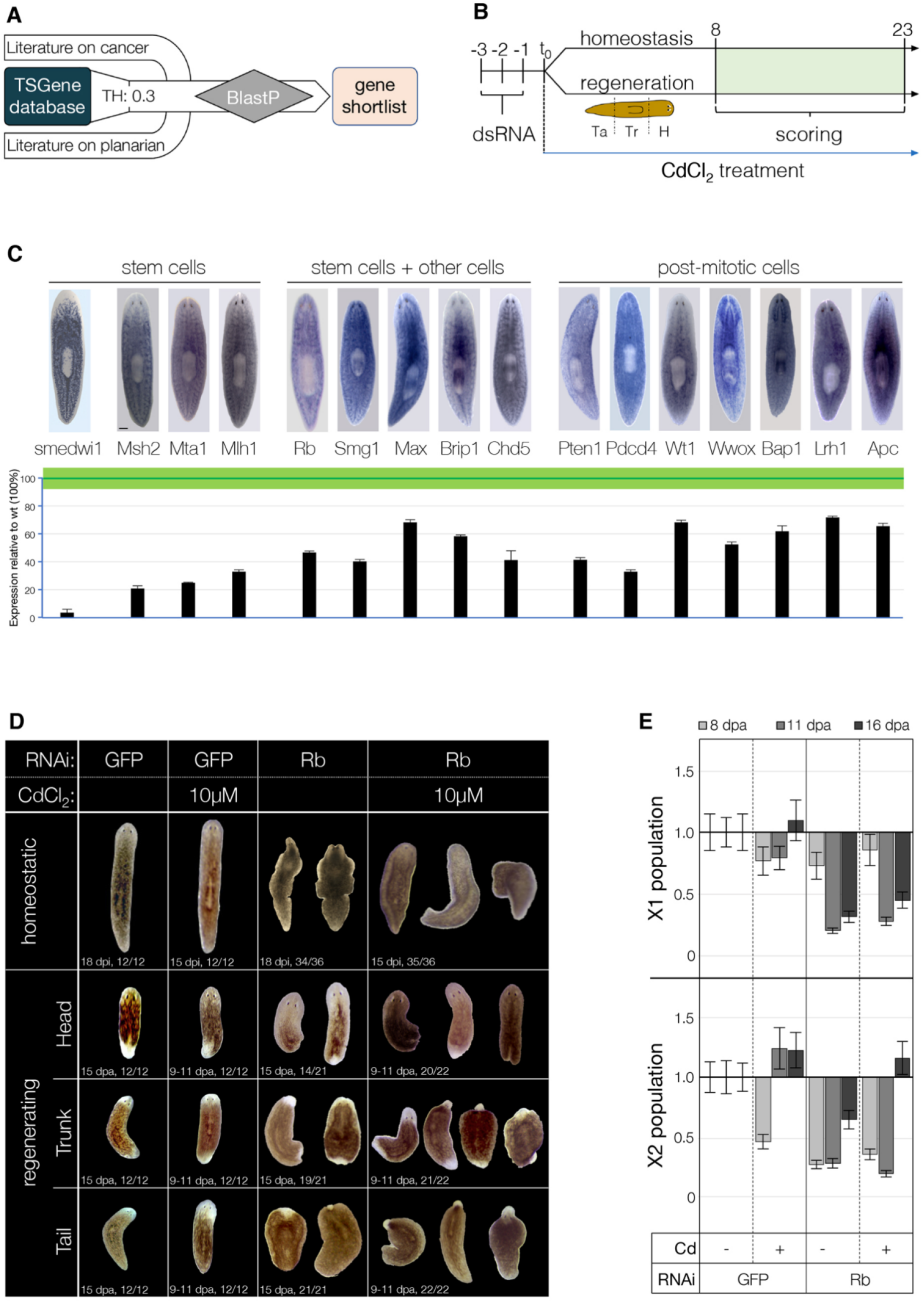
**TSGs in the planarian *S. mediterranea*.** (A) The TSG candidate shortlist was generated starting from the TSGene 2.0 database and integrated with the relevant literature on cancer. Known planarian TSGs were also considered. Planarian TSG homologs were searched on the SmedGD draft 3.1 using BLASTp. (B) Putative planarian TSGs were functionally screened by means of RNAi, as shown in the schematic. (C) The expression pattern of each candidate TSG was evaluated by whole-mount *in situ* hybridization (WISH) in wild-type animals (upper panel) and qPCR (lower panel); the green line represents mean±s.e.m. in wild-type animals (*n*≥3), black bars represent the expression in the irradiated animals (60 Gy; *n*≥3). Smedwi1 WISH/qPCR given as a stem cell-specific reference. Scale bar: 500 μm. (D) In the presence of Cd, the Rb(RNAi) phenotype was exacerbated in both homeostatic (upper row) and regenerating (lower rows) animals. As previously described, regeneration was largely impaired in Rb(RNAi) fragments. The presence of Cd increased the incidence of the regeneration phenotype (63/66 vs 54/63), accelerated its onset (9-11 vs 15 dpa) and worsened its severity. (E) The FACS fractions containing stem cells [X1 (upper chart) and X2 (lower chart)] were also quantitatively assessed by means of flow cytometry in regenerating animals at 8, 11 and 16 dpa. Data represent the average of two (11 dpa) or three (6, 16 dpa) experiments. Animals used for the experiments were starved for 2 weeks prior to use and had a length of 6 mm. For WISH, a minimum of three animals were used.

We used the TSGene 2.0 database (https://bioinfo.uth.edu/TSGene/index.html) (Zhao et al., 2013) to mine putative protein-coding TSGs out of the planarian genome. A shortlist of TSGs with a ratio of loss-of-function mutations over missense mutations ≥0.3 was further refined based on the available literature (Fig. 1A). Seventeen putative planarian TSG orthologs were found on the *S. mediterranea* genome draft (v3.1; Table S1), the function of which was investigated via RNAi (Fig. 1B). The expression patterns of Metastasis Associated 1 (*Mta1*, an epigenetic remodeler), MutL Homolog 1 and MutS Homolog 2 (*Mlh1* and *Msh2*, respectively, both DNA mismatch repair genes) (Hollenbach et al., 2011) are similar to that of *smedwi1*. Moreover, lethal irradiation (100 Gy) induced a downregulation of 70-80% compared with wild-type animals (Fig. 1C, bottom). The other TSGs have a less exclusive pattern of expression, yet nine of these were found downregulated in irradiated animals. Programmed Cell Death 4 (*Pdcd4*) and Phosphatase and Tensin Homolog 1 (*Pten1*) (Oviedo et al., 2008), involved in cell cycle regulation and apoptosis, showed progeny-like pattern and ≥60% downregulation in lethally irradiated animals (7 days post-irradiation) (Fig. 1C, bottom). Five are transcriptional modulators with gatekeeper function, namely MYC Associated Factor X (*Max*), Chromodomain Helicase DNA binding protein 4 (*Chd4*) (Scimone et al., 2010) and 5 (*Chd5*), Retinoblastoma Protein (*Rb*) (Zhu and Pearson, 2013) and BRCA1-Interacting Protein 1 (*Brip1*). The remaining two, Nonsense Mediated mRNA Decay-Associated PI3K-Related Kinase (*Smg1*) (González-Estévez et al., 2012) and *p53* (Pearson and Sanchez Alvarado, 2010) are caretaker genes. The other putative TSGs were mostly found expressed in post-mitotic cells – such as *Wt1*, *APC*, the pro-apoptotic modulator WW-domain containing Oxidoreductase (*Wwox*), the deubiquitinating enzyme BRCA1-Associated Protein 1 (*Bap1*) and the transcriptional regulator Liver Nuclear Receptor Homolog 1 (*Lrh1*) – and showed moderate downregulation after irradiation (Fig. 1C, bottom).

In the next step, the putative planarian TSGs were functionally screened via RNAi, in the presence or absence of the carcinogenic compound CdCl_2_ (Fig. 1B). Phenotypes were assessed morphologically, and by flow cytometry analysis, in both homeostatic and regenerating animals. KDs of *Smg1*, *Msh2*, *Pten1*, *p53*, *Chd4* and *Rb* were previously published (González-Estévez et al., 2012; Hollenbach et al., 2011; Oviedo et al., 2008; Pearson and Sanchez Alvarado, 2010; Scimone et al., 2010; Zhu and Pearson, 2013); however, exposure to the group 1A carcinogen Cd (IARC, 1993) allowed us to study their phenotypes in a carcinogenic context. Phenotypic abnormalities were detected in both homeostatic and regenerating animals after knocking down *Chd4*, *Rb* and *p53*. The KD of either *Pten1* or *Bcl2-3* produced a phenotype in regenerating animals only. Underlying the regeneration defects, we noticed that the loss of function of several TSGs (namely, *p53*, *Rb*, *Pten1*, *Chd4*, *Chd5*, *Max*, *Brip1* and *Bap1*) altered the balance between the X-ray-sensitive populations X1 and X2 (Fig. S1A), suggesting a role for these genes in the regulation of proliferation. In general, the phenotypes were more severe in combination with Cd exposure (Fig. 1D,E; Fig. S1B,C, Table S1). Dysplastic lesions and/or outgrowths were more often detected when either p53, Rb, Chd4 or Bcl2-3 RNAi animals were exposed to Cd. An overview of all results is provided in Table S1, out of which the most interesting phenotypes are discussed below.

The presence of Cd accelerated the onset of the Chd4(RNAi) phenotype in homeostatic animals of ∼3 days. In the absence of Cd, head regression and lateral constrictions appeared between 15 and 18 days after the last dsRNA injection (dpi) (*n*=34/36; Fig. S1B, Table S1). Animals exposed to Cd showed ventral curling and epidermal lesions before 15 dpi (*n*=34/36; Table S1), which are typical of stem cell deficiency (Zhu and Pearson, 2013). A similar acceleration of the onset of the phenotype was also observed in the exposed Chd4(RNAi)-regenerating fragments. Animals exposed to Cd displayed regeneration defects after 10±1 instead of 15±3 days post-amputation (dpa) (*n*=50/54; Fig. S1B, bottom rows, Table S1). Although both X1 and X2 populations were halved in Chd4(RNAi) fragments (52% and 57%, respectively), such a reduction was not further influenced by Cd (Fig. S1A).

Downregulation of the transcriptional corepressor gene *Rb* in homeostatic animals resulted in symmetric lateral constrictions (*n*=34/36 at 18 dpi), ventral curling and head narrowing (*n*=35/36 at 15 dpi; Fig. 1D, upper row). Homeostatic Rb(RNAi) animals exposed to Cd displayed depigmentation, loss of photoreceptors, head regression, abnormalities of the pharynx and ventral curling (*n*=35/36; Fig. 1D, upper row). During regeneration, Rb(RNAi) head and tail fragments failed to regenerate (*n*=54/63 at 15 dpa; Fig. 1D, lower rows), although blastema formation was initially unaffected. Cd exposure accelerated the onset of the phenotype of ∼5 days, making it more severe. Blastema formation was impaired and virtually all fragments failed to regenerate (*n*=63/66). Furthermore, dysplastic lesions, bloating and outgrowths – rarely observed in the absence of Cd – were observed at higher frequency. A reduction of both X1 (20% and 31% compared with GFP control at 11 and 16 dpa, respectively; Fig. 1E, upper chart) and X2 fractions (26%, 28% and 69% compared with GFP control at 8, 11 and 16 dpa, respectively; Fig. 1E, lower chart) was observed in Rb(RNAi) fragments. Intriguingly, exposure to Cd counteracted the reduction of the X2 subpopulation fraction in the long term (116% compared with GFP control at 16 dpa; Fig. 1E, lower chart).

### Cd induces oxidative stress and apoptosis in p53(RNAi) animals

**Fig. 2.**
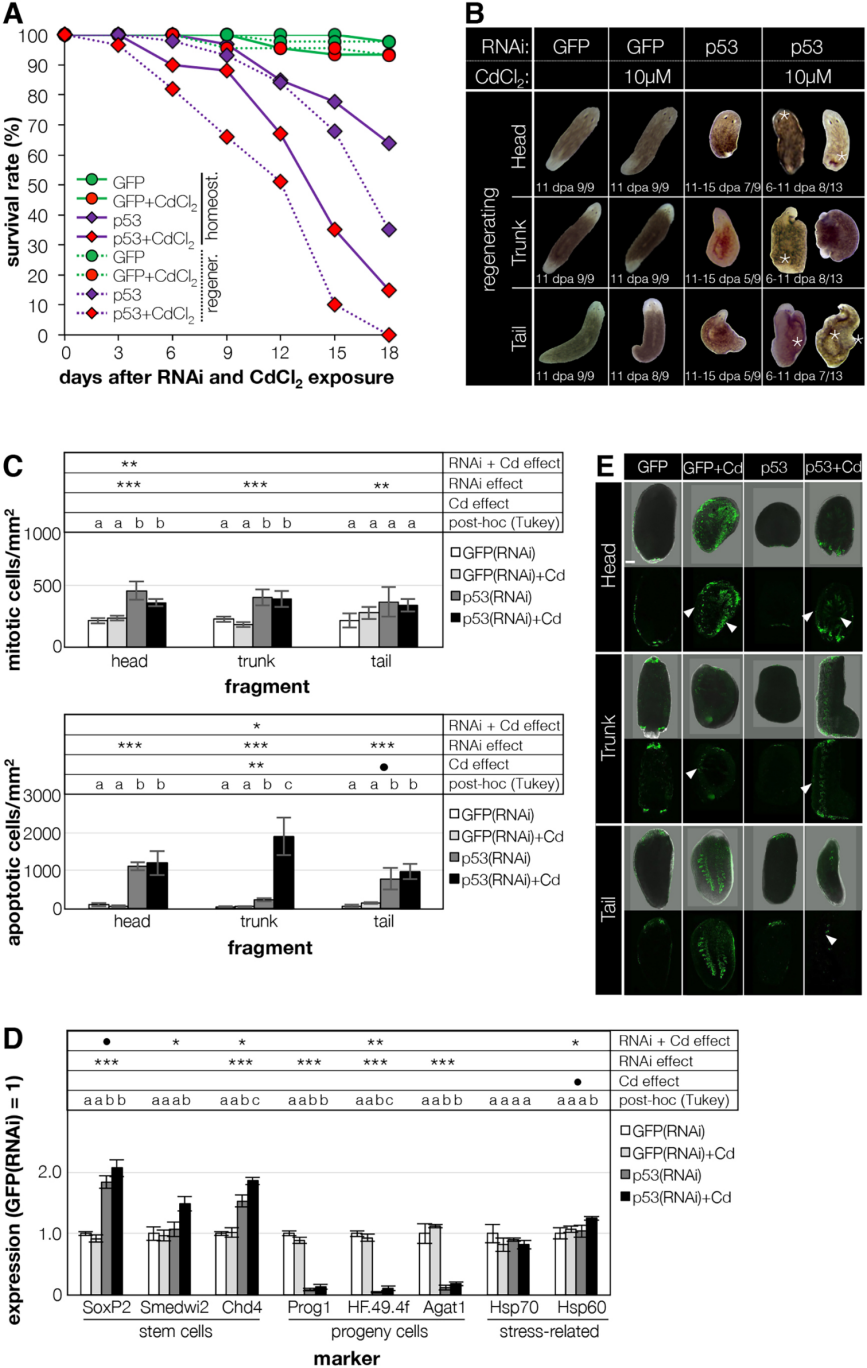
**Cd** **exacerbates the *p53*KD phenotype.** (A) Survival curve after *p53* KD in the presence or absence of the carcinogenic compound CdCl_2_. Data represent the average of two experiments (*n*=9-18 per group for regenerating animals; *n*=12 per group for homeostatic animals). (B) The presence of Cd increased the incidence of the p53(RNAi) phenotype and accelerated its onset. Dorsal outgrowths (asterisks) and bloating were only observed in Cd-treated fragments. Animals used for the experiments were starved for 2 weeks prior to use and had a length of 6 mm. (C) Mitotic (upper panel) and apoptotic (lower panel) figures were counted in regenerating fragments at 11 dpa, and normalized against the body area. Values are mean±s.e.m. of three to six biological replicates. (D) The expression of stem cell, progeny and stress-related genes was assessed via qPCR in regenerating head, trunk and tail fragments at 11 dpa (trunk fragments are shown as representative). Values represent the mean±s.e.m. of four to six biological replicates; expression is relative to that of GFP(RNAi) fragments (=1). Significance relative to the effect of RNAi alone, Cd alone or their combination was calculated with ANOVA and the results are shown in the table at the top of the chart: ^●^*P*≤0.1; *P≤0.05; **P≤0.01; ***P≤0.001; different letters in post hoc analysis indicate *P*≤0.05. Animals used for experiments were starved for 2 weeks and had a length of 6 mm. (E) The production of ROS was assessed in regenerating fragments at 3 hours post-amputation in the presence or absence of 50 µM CdCl_2_ via incubation with 25 µM carboxy-H2DFCA. Representative head, trunk and tail fragments are shown (top, bright field+fluorescence; bottom, fluorescence only) for the four experimental conditions tested. ROS were found at both amputation sites in all specimens, and along the intestinal branches in Cd-treated animals only [white arrowheads; *n*=0/18, 20/33, 0/15 and 18/24 for GFP(RNAi), GFP(RNAi)+Cd, p53(RNAi) and p53(RNAi)+Cd, respectively]. Scale bar: 100 µm.

For an in-depth understanding on the function of planarian TSGs in a carcinogenic environment, we focused on the most frequently mutated TSG, p53, evaluating its phenotype at the physiological, cellular and molecular levels, both in homeostasis and during regeneration. Downregulation of *p53* led to defects in tissue homeostasis, characterized by head regression, ventral curling and symmetrical lateral constrictions (Pearson and Sanchez Alvarado, 2010). In regenerating animals, the KD of *Smed-p53* resulted in a more complex phenotype. The majority of the fragments died between 11 and 17 dpa (Fig. 2A). Regeneration was severely impaired, although a blastema could form, and the differentiation of a hypotrophic head was observed, especially in trunk and tail fragments (Fig. 2B). Bloating, likely the consequence of edema formation, was sometimes observed in head fragments (data not shown). An additional exposure to Cd accelerated the onset of the observed phenotype (homeostatic, 11-15 vs 15-20 dpi; regenerating, 6-11 vs 11-15 dpa) and led to the development of dysplastic lesions, more evident in regenerating animals. When exposed to Cd, Smed-p53(RNAi) animals could not regenerate and occasionally displayed bloating and outgrowths (asterisks in Fig. 2B). At 17 dpa, all Cd-exposed fragments died (*n*=51/51), while 36% of the unexposed fragments survived (*n*=14/39; Fig. 2A). Notably, Cd alone did not increase lethality in control GFP(RNAi) animals.

We then assessed cellular behavior in p53(RNAi) regenerating animals at the onset of the phenotype (11 dpa), in the presence of Cd, as we wanted to trigger both cancer and regeneration simultaneously. Most stem cell-related effects were induced by *p53* KD; an additional Cd-related effect was mostly visible in the apoptotic burst. Specifically, in p53(RNAi) regenerating animals, mitoses were twice as many as in the respective GFP(RNAi) control (2.1-fold in head fragments, *P*≤0.001; 1.8-fold in trunk fragments, *P*≤0.001; 1.7-fold in tail fragments, *P*≤0.01) (Fig. 2C, upper panel; Fig. S2A). Proliferation was not further affected by exposure to Cd, whereas apoptosis was. In trunk fragments, the KD of *p53* induced a 6-fold increment in the apoptotic cell number (*P*≤0.001); Cd exposure induced a supplementary increase of almost 50-fold (*P*≤0.001) (Fig. 2C, lower; Fig. S2B). In the next step, the stem and progeny cell populations were assessed by fluorescence *in situ* hybridization (FISH), and the expression of stem cells, progeny and stress-related markers was quantified by quantitative polymerase chain reaction (qPCR). Neither *p53* KD, nor the presence of Cd, altered the number and the distribution of smedwi1^+^ (Fig. S2C, left column). Prog1^+^ cells, however, appeared to be reduced in p53(RNAi) animals, especially in the presence of Cd (*n*=3/4; Fig. S2C, right column). Cd-induced effects were barely measurable only for *Hsp60* expression [107% and 125% in GFP(RNAi)+Cd and in p53(RNAi)+Cd, respectively, *P*≤0.1]. KD of *p53* had a strong effect on the expression of stem and progeny cell markers. Both stem cell markers, *SoxP2* and *Chd4*, were upregulated (184% and 153%, respectively, *P*≤0.001), while all progeny markers showed a robust reduction of ∼10-fold (*P*≤0.001) (Fig. 2D). These data are aligned with the *p53* early phenotype, previously described (Pearson and Sanchez Alvarado, 2010). Five of eight markers showed Cd/p53 cumulative effect, although this was not always statistically significant. All the stem cell markers tested were found upregulated in p53(RNAi) fragments exposed to Cd [*SoxP2*, 123%, *P*≤0.1; *Smedwi2*, 142%, *P*≤0.05; *Chd4*, 133%, *P*≤0.05, compared with p53(RNAi) fragments] (Fig. 2D). In spite of the p53-induced downregulation, *HF.49.4f* was also found to be upregulated as a consequence of Cd exposure (275%, *P*≤0.01). *Hsp60* displayed a modest Cd-induced upregulation as well (142%, *P*≤0.05).

Because Cd administration is known to rapidly (but transiently) induce the formation of reactive oxygen species (ROS), and these might influence cancer hallmarks, we monitored transient ROS formation in live fragments immediately after amputation, either in the presence or absence of Cd. When comparing conditions, we only detected an increased ROS production in the gastrovascular system (white arrowheads in Fig. 2E) of fragments treated with Cd. Such an effect was observed in all fragments, but it was stronger in trunk fragments [(7/11 fragments in the GFP(RNAi)+Cd group; 7/8 fragments in the p53(RNAi)+Cd group].

To better understand how fast-remodeling tissues respond to carcinogenic substances, we investigated the underlying changes triggered by Cd at the proteomic level.

### A proteomic screen revealed the effects of Cd exposure on the expression of stress- and tumor-related genes

**Fig. 3.**
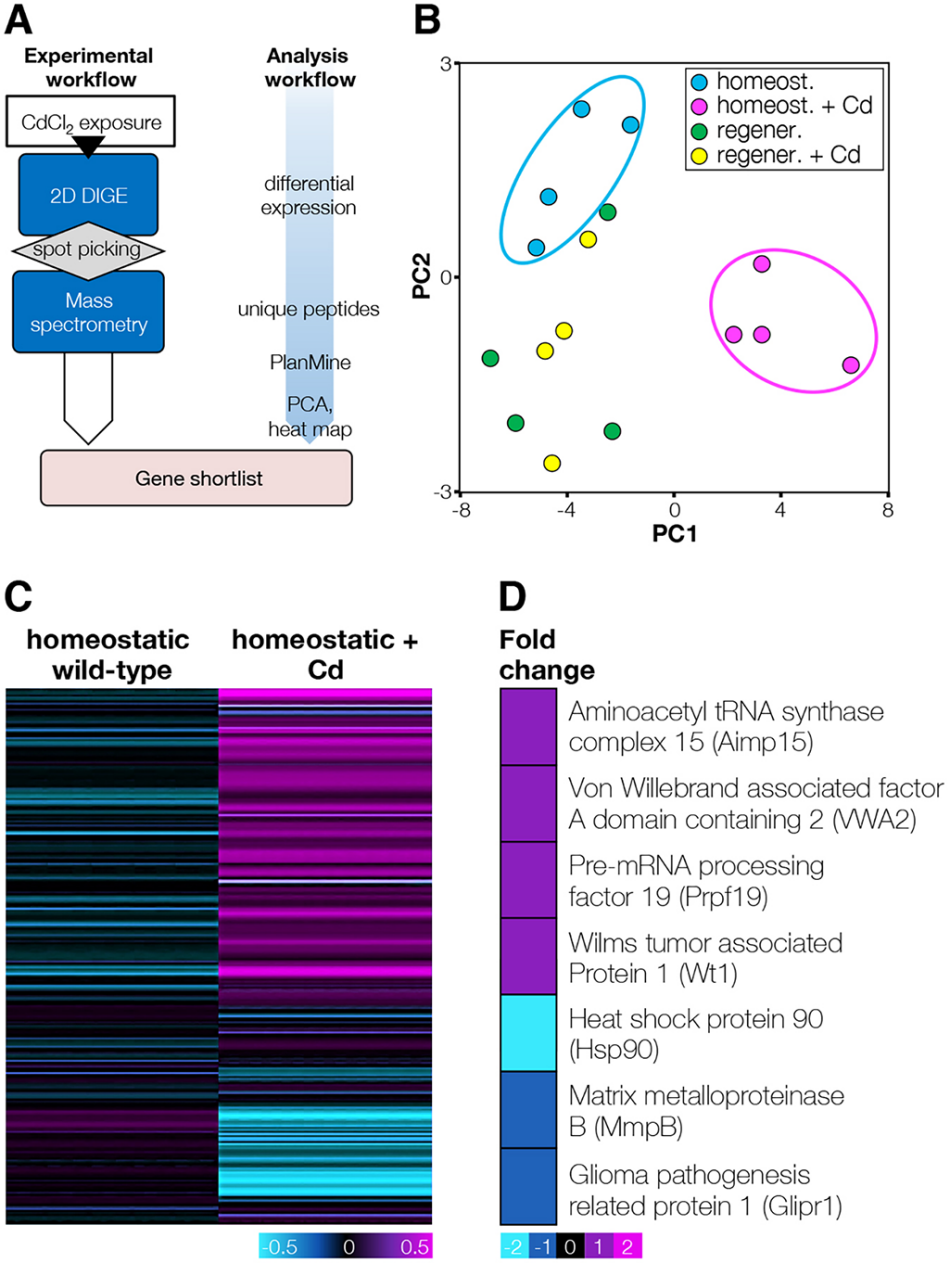
**Cadmium induces changes in the proteomic signature of homeostatic animals.** (A) Proteomic screen at a glance. (B) Principal component analysis of the proteomics samples. Four independent replicates were run for each of the conditions considered: homeostatic wild-type, homeostatic+Cd (14 days exposure), regenerating wild-type (7 dpa) and regenerating+Cd (7 dpa). Three clusters of samples were individuated corresponding to homeostatic wild-type, homeostatic Cd and regenerating (wild-type+Cd). (C) Uniquely identified by mass spectrometry, 172 proteins were found differentially expressed between homeostatic wild-type and homeostatic Cd samples and arranged in a heat map. (D) Detail of the generated heat map for ten proteins that are related to cancer. Fold changes are presented in a logarithmic scale.

Proteomes were analyzed in both regenerating and homeostatic animals (Fig. 3A). In order to focus on the effects induced by Cd to the stem cells, samples were taken when the proliferative peak was observed (i.e. after 1 week of exposure in regenerating animals and after 2 weeks of exposure in homeostatic animals). Altogether, 476 unique protein spots were identified as differentially expressed in at least one of the four experimental groups (difference≥±1.5-fold; *P*≤0.05) by two-dimensional difference gel electrophoresis (2D-DIGE). Principal component analysis indicated the Cd-exposed and unexposed homeostatic animals as the most divergent groups of samples, while all regenerating samples clustered together (Fig. 3B). This suggested either that regeneration covers the effects induced by Cd, or that regeneration and Cd trigger similar effects at the proteomic level. Based on fold changes in protein spot intensity of ≥±1.5 and proper matching with preparative spot maps, 251 spots were picked for protein identification by mass spectrometry. Of these, 215 were differentially expressed between Cd-exposed and unexposed homeostatic animals, and 33 were differentially expressed between Cd-exposed homeostatic and Cd-exposed regenerating animals. The difference between nonexposed and Cd-exposed regenerating planarians was limited to four spots only.

Liquid chromatography–tandem mass spectrometry (LC-MS/MS) spectra were generated for the 251 differentially expressed protein spots. Two protein databases were simultaneously searched, which resulted in the identification of one or more unique peptides corresponding to 172 unique proteins (ProteomeXchange dataset PXD009852). The heat map generated by hierarchical clustering showed that among these 172 proteins, 75% were upregulated and 25% downregulated in homeostatic animals treated with Cd, compared with their respective controls (Fig. 3C). Among the upregulated proteins, we found cellular stress response factors, such as Von Willebrand factor A domain-containing 2 (VWA2, +1.7-fold, *P*≤0.005) and Aminoacyl tRNA synthase complex (AIMP1, +1.8-fold, *P*≤0.001). Among the downregulated proteins, we found two proteins with tumor suppression function (Fig. 3D), Glioma Pathogenesis-Related Protein 1 (GLIPR1, −1.7-fold; *P*≤0.01) and Smed-MMPB (−1.8-fold; *P*≤0.05); interestingly, MMPB is one of the four proteins that were downregulated in Cd-exposed regenerating animals (−1.6-fold; *P*≤0.05). *Smed-MmpB* is the planarian ortholog of MMP19 (Isolani et al., 2013) (Fig. S3A). MMP19 is a key modulator in many cellular and developmental processes such as cell migration and transformation; it was reported to act as both tumor promotor and tumor suppressor (Noel et al., 2012). Remarkably, HSP90, a chaperone that among its client proteins has both proto-oncogenes, such as *Oct4* (Bradley et al., 2012), and TSGs, such as *p53* (Walerych et al., 2004), was one of the most downregulated proteins in Cd-exposed animals (Fig. 3D).

All the above-mentioned genes were knocked down. Following the KD of *Smed-Glipr1* and *Smed-MmpB*, planarians developed dysplastic lesions; therefore, the two genes were further investigated.

### *Smed-Glipr1* and *Smed-MmpB* act as TSGs

**Fig. 4.**
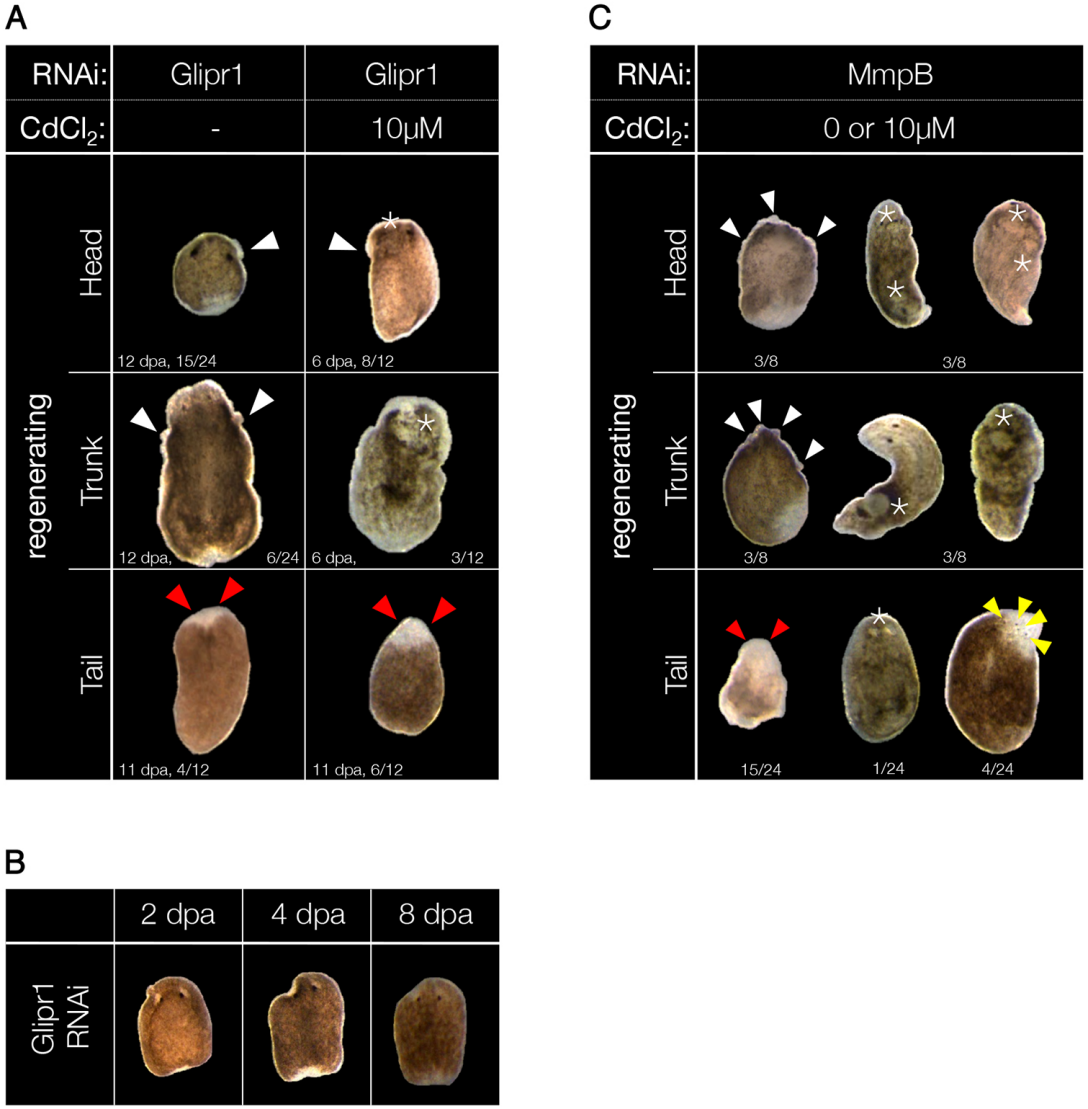
***Glipr1* and *MmpB* act as** **TSGs.** (A) Morphological changes associated with the downregulation of *Glipr1* during regeneration are shown. The most frequently associated defects were epidermal blisters (white arrowheads) and lack of photoreceptors (red arrowheads). Exposure to Cd caused depigmentation, bloating and the development of dorsal outgrowths, especially in head and trunk fragments (asterisks). (B) Smaller outgrowths developed in Glipr1(RNAi) animals also in the absence of Cd; however, these outgrowths could be resorbed (*n*=7/21) and healed by the animal (*n*=4/7 at 21 dpa). (C) The *Smed-MmpB* phenotype was not affected by Cd exposure. Typical lesions associated with *MmpB* KD were epidermal blisters (white arrowheads), lack of photoreceptors (red arrowheads), supernumerary photoreceptors (yellow arrowheads) and large outgrowths (asterisks). Animals used for the experiments were starved for 2 weeks prior to use and had a length of 6 mm.

The KD of *Smed-Glipr1* resulted in the development of small epidermal blisters (white arrowheads in Fig. 4A) and tumor-like outgrowths (asterisks in Fig. 4A) in both unexposed and Cd-exposed regenerating animals, especially in head (*n*=15/24) and trunk (*n*=6/24) fragments. Although none of the Smed-Glipr1(RNAi) tail fragments developed outgrowths, they also died, starting at 11 dpa onwards. Cd treatment accelerated the onset of the phenotype, increasing both the frequency and the severity of the lesions. In some cases, tail fragments failed to regenerate photoreceptors (red arrowheads in Fig. 4A). In general, malformations were more abundant in head fragments compared with other body fragments. A consistent portion of the fragments that developed outgrowths was able to clear them, regenerating the damaged tissues as after wounding (*n*=7/21; Fig. 4B). Four of seven animals that cleared the outgrowths survived to the end of the screening period, while all the fragments that failed to clear the outgrowths died (*n*=14/21).

Planarian MMPB was recently described as a putative membrane-type MMP (MT-MMP), owing to the presence of a transmembrane domain (TMD) in its *N*-terminus (Isolani et al., 2013). According to TMHMM (http://www.cbs.dtu.dk/services/TMHMM/), neither Smed-MMPB, nor its human ortholog MMP19 possess a TMD, which is otherwise detected in human MT-MMP24 (Fig. S3B). The identification of the projected conserved domains in these MMPs by InterProScan (https://www.ebi.ac.uk/interpro/) suggested that the N-terminus signal peptide was erroneously mistaken for a transmembrane domain, which in MT-MMPs always localizes to the C-terminus (Cao et al., 1995) (Fig. S3C).

The KD of *Smed-MmpB* produced a phenotype similar to that of *Smed-Glipr1*, characterized by the presence of both small epidermal blisters (*n*=6/24; white arrowheads in Fig. 4C) and large outgrowths (*n*=7/24; asterisks in Fig. 4C). However, the presence of outgrowths strongly varied among experiments, even though KD efficiency did not vary accordingly. Similarly to what was observed in Smed-Glipr1(RNAi) animals, these lesions mainly occurred in head and trunk fragments, and affected both Cd-exposed and unexposed animals alike. Occasionally, bloating of the animal occurred. The most frequent defect observed in tail fragments was the lack of regeneration of one or both photoreceptors (*n*=15/24; red arrowheads in Fig. 4C). Supernumerary/fragmented photoreceptors were also occasionally observed (yellow arrowheads in Fig. 4C). The failure to regenerate the photoreceptors also afflicted, although to a lesser extent, trunk fragments (*n*=3/8). Differently to what was observed after *Glipr1* KD, exposure to Cd affected neither the frequency nor the severity of the lesions developed in Smed-MmpB(RNAi) animals. In fact, the presence of Cd delayed the onset of the *MmpB* phenotype, which emerged after 10 dpa in unexposed fragments and after 23 dpa in Cd-exposed ones.

Altogether, our data suggest that both GLIPR1 and MMPB have tumor suppressor function in *S. mediterranea*, as their downregulation led to the development of macroscopic dysplastic lesions affecting the entire body of the animal. These lesions sometimes developed into large outgrowths similar to those found after KD of *Pten1/2*, *p53* and *Rb* (Oviedo et al., 2008; Pearson and Sanchez Alvarado, 2010; Zhu and Pearson, 2013). The presence of Cd highlighted profound differences in the function of the two genes. In the case of *Glipr1* KD, Cd accelerated the onset of the phenotype and enhanced its severity, while in the case of *MmpB* KD, Cd slowed down the onset of the phenotype, without affecting its severity.

### MmpB(RNAi) planarians develop epidermal blisters with proliferating stem cells

**Fig. 5.**
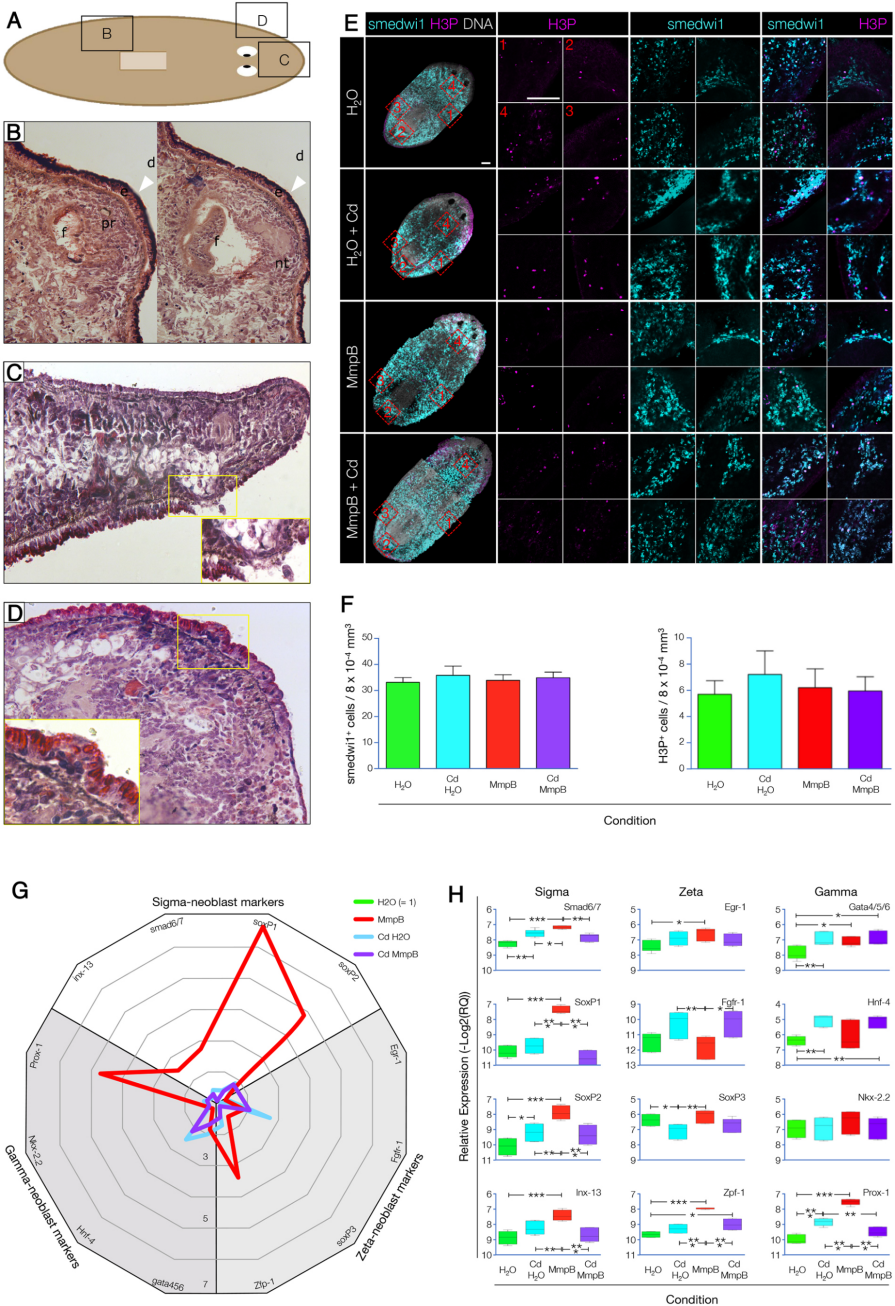
**Smed-MmpB KD promotes tissue invasion and tumor formation.** (A) Localization of the lesions shown in B and C. (B) Sagittal section of the dorsal posterior region stained with H&E. The large outgrowth (white arrowheads) contained ectopic structures such as photoreceptors (pr), neural tissue (nt) and a pharyngeal pocket (f), resembling a teratoma (d, dorsal). (C) Sagittal section of the median anterior region stained with H&E. The lesions observed were characterized by the displacement of gastrointestinal tissue that ruptured the epidermal layer [magnification, 40× (inset, 100×)]. (D) Frontal section of the anterior region stained with H&E. Epidermal blisters accumulated in the region anterior to the pharynx and were characterized by the presence of small cells that crossed the basal membrane, without altering the epidermal layer [magnification, 40× (inset, 100×)]. (E) Body-wide distribution of smedwi1^+^ and H3P^+^ cells. Stitched maximum confocal projection of the whole animal imaged at 20× (left column). Magnifications of the lateral pre-pharyngeal (1), posterior back-stripe (2), lateral post-pharyngeal (3) and head/neck (4) areas, corresponding to the red frames shown in the left column, are presented in a clockwise arrangement. Scale bars: 100 µm. (F) No significant differences were found when the numbers of smedwi1^+^ or H3P^+^ cells were scored. (G) Radar chart showing the relative enrichment in the expression of subsets of sigma-, zeta- and gamma-neoblast markers in the four experimental conditions tested (*n*=3; H_2_O control=1 and collapsed to the center). (H) Boxplots showing the significant differences in the relative expression of the 12 markers tested. *P≤0.05; **P≤0.01; ***P≤0.0001.

In order to understand MMPB function at the cellular and molecular levels, we investigated the landmarks of the *MmpB* phenotype (outgrowths, lesions and epidermal blisters) for histological evidence (Fig. 5A). When a large dorsal outgrowth was sectioned and stained with Hematoxylin and Eosin (H&E), it revealed the presence of differentiated tissues inside. We could observe neural cells, pigmented cells organized in an optic cup and a small ectopic pharynx (Fig. 5B). Although the architecture of tissues and organs inside the outgrowth appeared normal, their organization was seemingly casual. Therefore, *MmpB* outgrowths differed from those originated as a result of axis duplication (Kato et al., 1999) as well as from those developed after EGFR1 KD (Fraguas et al., 2011); rather, they resembled the teratoma-like structures that were observed after low-dose p53(RNAi) (Pearson and Sanchez Alvarado, 2010). The small lesions that affected MmpB(RNAi) animals with anterior-posterior gradient were also investigated. Transverse sections stained with H&E revealed that they owed largely to the abnormal expansion of the gut, which squeezed apart the parenchyma, broke through the basal lamina and burst the epidermal layer below (Fig. 5C and its inset). Epidermal blisters, the third landmark of *MmpB* KD, did not seem to be the initial step of the aforementioned lesions. Nor did they look similar to the epidermal dysplasia observed after *Smed-Mmp1* KD, in which a pluristratified epidermis was observed (Isolani et al., 2013). Rather, *MmpB* blisters resulted from the local accumulation of small cells beneath the epidermis (Fig. 5D). These cells had a sparse cytoplasm, high nucleus-to-cytoplasm ratio and did not contribute to the multiple stratification of the epidermal layer, but accumulated in small pouches (insert in Fig. 5D) with a translucent look (arrowheads in Fig. 4C). In order to understand the origin of the dysplastic lesions observed, we looked at the distribution of the stem cell population body-wide, which was unaltered among the four experimental conditions tested (Fig. 5E, left column). Four quadrants were considered and the numbers of stem cells (smedwi1^+^) and actively proliferating cells [serine 10-phosphorylated histone 3-positive (H3P^+^)] did not show any significant difference (Fig. 5E,F). Neoblast subpopulations have recently been described (sigma-, zeta- and gamma-neoblasts) (van Wolfswinkel et al., 2014), defined by specific subsets of genes enriched in each subclass. We quantified the expression of these markers by qPCR, and found that MmpB(RNAi) animals overexpress 6/12 markers compared with all other three experimental conditions. These were the four sigma-neoblast markers (smad6/7, +2.7-fold change; SoxP1, +6.5-fold change; SoP2, +4.3-fold change; inx-13, +2.5-fold change; Fig. 5G,H, left column), the zeta marker Zfp-1 (+2.7-fold change; Fig. 5G,H, middle column) and the gamma marker Prox-1 (+4.8-fold change; Fig. 5G,H, right column).

**Fig. 6.**
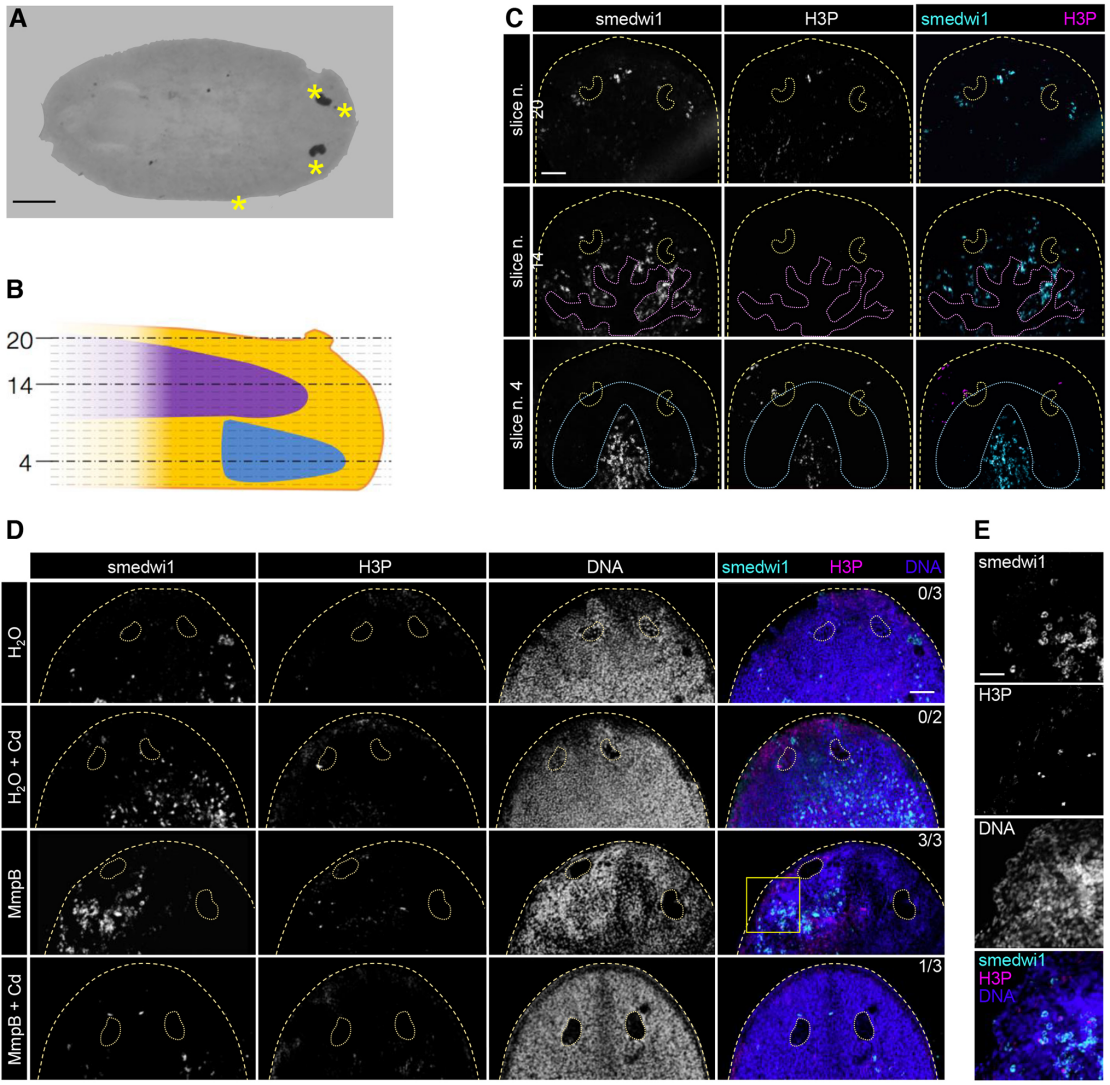
**MmpB-dependent skin blisters are populated of proliferating, smedwi1^+^ stem cells.** (A) Skin blisters were normally found in the cephalic region. Scale bar: 300 µm. (B) Confocal acquisition of the sample in A. (C) Confocal slices of smedwi1/H3P double WISH/immunohistochemistry (IHC). Slice numbers 4 (brain level), 14 (intestine level) and 20 (dorsal epidermis level) are shown. In confocal slice 20, three small clusters of smedwi1^+^ cells are visible around the photoreceptors; the central one has two H3P^+^ cells (upper row). Scale bar: 100 µm. (D) Smedwi1^+^/H3P^+^ skin blisters were only found in MmpB(RNAi) animals, either in the presence (*n*=1/3) or absence (*n*=3/3) of Cd. Images are single confocal slices acquired at the dorsal epidermal level. Numbers in the top-right corners of images indicate animals with smedwi1^+^/H3P^+^ skin blisters for each of the four conditions considered. Scale bar: 100 µm. (E) A 63× magnification of the skin blister in D (yellow box). Several smedwi1^+^ cells are visible, six of which are also H3P^+^. Scale bar: 40 µm. Yellow dashed lines in C and D demarcate the animal edge and photoreceptors; purple dashed lines in C demarcate the anterior intestinal branch; blue dashed lines in C demarcate the brain.

Eventually, we characterized the cells that populate the small epidermal blisters that affected one quarter of the MmpB(RNAi) animals (*n*=6/24). Affected animals developed one to four blisters, which were mainly found in the anterior, either at the lateral edges or on the dorsal epidermis (Fig. 6A). The small cells found in the blisters morphologically resembled the neoblasts, the planarian stem cells. Therefore, we assessed both the expression of the stem cell marker smedwi1 and the presence of H3P, a marker of the G2/M phase. In order to exclude false positives, confocal slices were taken covering the entire thickness of the specimen (Fig. 6B). In each of the analyzed blisters (*n*=12), we found at least four smedwi1^+^ cells (Fig. 6C), usually more (9.5±2.7). Within the blisters, some smedwi1^+^ cells were also positive for H3P (2.0±0.8). The majority of smedwi1^+^/H3P^+^ cells were found in the blisters of MmpB(RNAi) animals not exposed to Cd (*n*=3/3; Fig. 6D); however, it is possible that the delay observed in the onset of the phenotype exerted by Cd also delayed the formation of the stem cell blisters. In large blisters, up to 25 smedwi1^+^ cells and up to six smedwi1^+^/H3P^+^ cells were detected (Fig. 6E).

## DISCUSSION

TSGs, such as Smg1, p53, Rb and PTEN, are functionally involved in the regeneration process (González-Estévez et al., 2012; Hall et al., 1986; Oviedo et al., 2008; Seifert and Voss, 2013). This is likely to be one of the reasons why highly regenerative tissues and organisms show great plasticity against tumorigenic malformations (Stevens et al., 2016). To further explore the underlying relationships, we simultaneously triggered regeneration and carcinogenesis in the planarian *S. mediterranea*. Carcinogenesis was induced via chemical exposure to the carcinogen CdCl_2_, and its effects were evaluated in both homeostatic and regenerating animals. In the first part of our study, selected TSGs were functionally validated. In the second part, proteomes of the different conditions were analyzed in a nontargeted approach.

Known and putative planarian TSGs were selectively knocked down in animals exposed to Cd. Of the 18 genes tested, the KD of *p53*, *Rb*, *Chd4*, *Pten1*, *Bap1*, *Brip1* and *Bcl2-3* produced a stem-cell-depletion phenotype (e.g. ventral curling, head regression, regeneration defects) (Figs 1 and 2; Fig. S1, Table S1). The presence of Cd accelerated the onset of most of the phenotypes described and increased both the severity of the lesions and the number of animals affected (Figs 1 and 2; Table S1). This suggests that a fully operative stem cell machinery is an indispensable first-line defense against the carcinogenic drift. Normally, the functional failure of these genes coupled to Cd-induced stress hastens carcinogenesis (Hartwig, 2013; Méplan et al., 1999), but the development of dysplastic lesions (e.g. epidermal blisters, large outgrowths) was rare, even in Cd-exposed RNAi planarians. Among TSGs, *p53* is the most frequently mutated gene in cancer; it is involved in self-renewal (Brandner, 2010; Lin et al., 2005; van der Laan et al., 2013) and its function has been extensively characterized in planarians (Pearson and Sanchez Alvarado, 2010). After further investigation, our findings confirmed that in a carcinogenic environment, *Smed-p53* also has a broad range of functions. *Smed-p53*(RNAi) fragments were able to initiate regeneration, as initially *p53* KD does not induce a decrease in the stem cell population. On the contrary, until a clear phenotype emerged (from 11 dpa onwards), data indicate that both the number of mitoses (Pearson and Sanchez Alvarado, 2010) (Fig. 2C, upper panel) and the expression of stem cell-specific markers (Fig. 2D) increased as a consequence of *p53* KD.

The number of apoptotic cells was also significantly higher in p53(RNAi) animals (Fig. 2C, lower panel), which counteracted the increased mitotic rate, levelling the total stem cell number and affecting the post-mitotic progeny, as for the decreased expression of the epidermal progeny markers (Fig. 2D). Later (13-15 dpa), a typical stem cell-deficient phenotype emerged, as described (Pearson and Sanchez Alvarado, 2010); yet, animals did not show any evidence of carcinogenic lesions. Intriguingly, the presence of Cd not only accelerated the onset of the *p53* phenotype and enhanced its severity (Fig. 2A-C), but also caused a tumorigenic drift, with the development of dorsal outgrowths and epidermal lesions (Fig. 2B), although limited to few individuals. The high levels of ROS found in the gastrovascular system of Cd-exposed regenerating animals could be involved in the development of the outgrowths observed (Fig. 2B,E). We hypothesized that this redox unbalance was also responsible for the massive apoptotic wave observed in the p53(RNAi) animals exposed to Cd (Fig. 2C). These findings are in line with the role of *p53* in regulating proliferation and cell survival.

Different species of planarians showed a broad range of outcomes when exposed to Cd. *Dugesia dorotocephala* developed benign tumors that turned malignant upon co-administration of tumor promoter 12-O-tetradecanoylphorbol-13-acetate (TPA) (Hall et al., 1986). *Dugesia tigrina*, on the other hand, developed multiple malignancies upon exposure to Cd, which reduced in number when TPA was co-administered (Voura et al., 2017). According to our data, Cd alone did not induce tumors in *S. mediterranea*, which emerged only if a certain TSG (e.g. p53, *Rb*) was knocked down (Figs 1 and 2; Fig. S1). Even then, a limited number of animals was affected. To our understanding, different planarian species developed different mechanisms to cope with a carcinogenic environment. To unravel the *S. mediterranea* way, a proteomic screen was run to compare homeostatic and regenerating animals in the presence or absence of Cd. Remarkably, we could not find substantial differences between Cd-exposed and unexposed regenerating fragments (Fig. 3B), although regenerating fragments exhibited a sensitivity to Cd higher than that of homeostatic animals. Once more, this evidence supports the idea that triggers as different as the loss of tissue and exposure to carcinogenic compounds could lead to outcomes as different as regeneration and cancer, but through a common path (i.e. inflammation) (Karin and Clevers, 2016). A clear Cd-dependent difference was found for homeostatic animals. Altogether, 215 proteins showed differential abundances. Among those, several have literature relevance in carcinogenic processes (Fig. 3D), and were therefore functionally tested via RNAi. The KD of two of these proteins, namely GLIPR1 and MMPB, resulted in the induction of dysplastic lesions (Fig. 4).

Although a known target of *p53*, *Glipr1* also has a p53-independent tumor suppressor function, as its misregulation is associated to colorectal cancer, myeloma and prostate cancer (Aytekin et al., 2010; Li et al., 2008; Tam et al., 2010). We observed that 29% of the regenerating fragments displayed tumor-like protrusions (Hanahan and Weinberg, 2011), mostly epidermal blisters (Fig. 4A). The incidence of these lesions had an anterior-posterior gradient (head: trunk: tail=63%: 25%: 0%) and showed up in both Cd-exposed and unexposed Glipr1(RNAi) fragments. The tissue-specific function of this gene, and the dual role played as either tumor promoter or tumor suppressor (Awasthi et al., 2013; Thompson, 2010), could explain the gradient found, while the fact that protrusions appeared under both control and carcinogenic conditions, suggests that it has a regulatory function in activating the proliferation response following amputation. Furthermore, GLIPR1 exerts its pro-apoptotic activity through the JNK signaling cascade (López-Otín et al., 2009), in the presence of ROS. This could explain why we found cancerous lesions in regenerating animals, where JNK is active, and not in homeostatic animals, where JNK is mostly inactive (Almuedo-Castillo et al., 2014).

The second gene for which KD produced dysplastic lesions was *Smed-MmpB*. It is the planarian ortholog of vertebrate MMP19, a zinc-dependent matrix metalloproteinase which, amongst others, has an essential function in scar-free regeneration processes (Huang et al., 2014; Seifert et al., 2012). Because MMP-mediated degradation of the ECM is an important event in the regulation of cancer cell survival, transformation and migration, MMPs were generally regarded as tumor promoter genes. Over the last decade, however, tumor suppression functions have paradoxically been proposed for a few members of the MMP family, such as MMP19 (Chan et al., 2011; Hynes, 2009). Differently from what was previously reported (Isolani et al., 2013), we observed dysplastic lesions (epidermal blisters, epidermal lesions and large outgrowths) (Fig. 4C) emerging in Smed-MmpB(RNAi) regenerating animals, both Cd-exposed and unexposed. Although the frequency of tumor formation varied, in some experiments *Smed-MmpB* KD induced tumors in as many as 39% of the regenerating fragments. Histological analysis showed that both blisters and outgrowths stemmed from an accumulation of hyperplastic cells. In the case of the large outgrowths, these cells differentiated ectopically into nerve tissue, photoreceptors and even a small pharynx, altogether resulting in the formation of a teratoma-like structure (Fig. 5B). These tumors progressively spread throughout the entire body, and caused the death of the animal. Epidermal blisters formed as a consequence of the accumulation of undifferentiated cells under the epidermal layer. The disruption of the basement membrane (inset in Fig. 5D) indicated tissue invasion, which is a characteristic of malignancy. Not surprisingly, the large majority of the cells found in the blisters were smedwi1^+^; what we found remarkable, was their high mitotic rate. On average, one of 4.75 smedwi1^+^ cells was also positive for the mitotic marker H3P (Fig. 6; Fig. S4). Tumors were only observed after Smed-MmpB(RNAi) fragments had completed regeneration (i.e. from 10 dpa onwards). This could be caused by the fact that small developing tumors went unnoticed, but might also suggest a dual role for Smed-MMPB, according to the state of the microenvironment. During regeneration, Smed-MMPB influences tissue remodeling, promoting the stem cell-based restoration of the homeostasis, as shown in vertebrates (George and Dwivedi, 2004). This could explain the regeneration defects observed after Smed-MmpB KD. Once regeneration is complete, we hypothesized that Smed-MMPB preserves the stem cell niche, avoiding uncontrolled proliferation; its lack promotes the onset of a dysplastic phenotype, as it was shown in ‘aged’ microenvironments, which are more likely to develop tumors, independently of the number of mutations accumulated (DeGregori, 2017).

In conclusion, our data support the hypothesis that *S.* *mediterranea* is more resilient than other planarian species towards carcinogen-induced tumor formation, as previously shown (Plusquin et al., 2012). Underlying this plasticity, we observed the involvement of several TSGs, either known or novel, for which loss of function, in combination with exposure to Cd, destabilized the stem cell compartment, triggering tumor formation. Despite the fact that Cd alone was not able to induce tumorigenesis in *S. mediterranea*, its presence in the maintenance medium resulted in the differential regulation of 172 proteins in homeostatic animals, as revealed by 2D-DIGE. Among those, many stress- and tumor-related genes were found up- or downregulated. The proteomic data also stressed the importance of the stem cell niche: surprisingly, a proliferation-prone, tightly regulated environment such as the amputated planarian seemed less subject to Cd-induced changes of the proteome, while the homeostatic animals showed a higher sensitivity to the carcinogenic compound at the molecular level. The loss of function of two tumor-related proteins that were found downregulated in Cd-exposed planarians induced tumor formation in regenerating animals. *Smed-MmpB* KD, in particular, induced different types of protrusions, containing actively proliferating smedwi1^+^ cells. This suggests a control exerted by the ECM on the stem cell compartment, which oversees the migration of the stem cells and prevents their ectopic proliferation. These findings are in line with the involvement of similar mechanisms in both regeneration and tumorigenesis in vertebrate model systems. Although a further effort is needed to unravel how the integrated network of TSGs prevents stem cell transformation and tumorigenesis, our work pinpoints that planarian tumors might originate from the ectopic, uncontrolled proliferation of the ordinary stem cells, and that TSGs and the stem cell niche have a central role in their transformation.

## MATERIALS AND METHODS

### Planarian husbandry and CdCl_2_ exposure

Planarians (asexual strain of *S.* *mediterranea*) were kept in the darkness at 19-20°C, in planarian artificial medium (PAM) and fed twice a week with calf liver. Animals were starved for 2 weeks before each experiment, and were not fed during the experiments. A 1 M CdCl_2_ stock solution was prepared and freshly diluted to 10 µM CdCl_2_ in PAM before use. To regenerating animals, Cd was administered 30 min after amputation.

### Planarian TSGs *in silico* mining

The current collection of human TSGs was acquired from the TSGene 2.0 database (https://bioinfo.uth.edu/TSGene/index.html) (Zhao et al., 2013). Mining and selection of putative planarian TSGs was operated as illustrated in Fig. 1A. Briefly, a ratio of loss-of-function mutations over missense mutations ≥0.3 was applied to the complete TSG list; the resulting genes were searched on the SmedGD v3.1 by BLASTp (Schneider et al., 2012).

### RNAi and morphological screen

Gene-specific dsRNAs were generated with the T7 Ribomax Express RNAi system (Promega). RNAi primers are listed in Table S2. Three pulses of 32 nl each of 1 µg/µl dsRNA during three successive days were injected in the prepharyngeal area of the gastrovascular system with a Nanoject II (Drummond Scientific, Thermo Fisher Scientific, Dreieich, Germany). Experiments were conducted and assessed as depicted in Fig. 1B. For each condition, a minimum of 16 homeostatic and 24 regenerating animals were used. Between day 8 and day 23, specimens were collected for downstream analysis.

### WISH and IHC

WISH and IHC were performed as previously described (King and Newmark, 2013; Pearson et al., 2009; Reddien et al., 2005). Briefly, DIG-UTP- (Roche Diagnostics, Mannheim, Germany) labeled sense and antisense riboprobes were allowed to hybridize at 56°C for 20-40 h. Hybridized riboprobes were either detected via NBT/BCIP precipitation or fluorescence, using Anti-Digoxigenin-POD Fab fragments (1:100; Roche) anti-phospho-histone H3 (Ser10) antibody (1:1000, Millipore, Merck Chemicals, Darmstadt, Germany) and Alexa Fluor 647-conjugated goat anti-rabbit IgG (1:1000; Life Technologies, Darmstadt, Germany) were used as primary and secondary antibodies to detect cells in G2-to-M phase. Hoechst 33342 (5 µg/ml; Thermo Fisher Scientific) was used for nuclear counterstaining. Samples were embedded in Aqua Poly/Mount (Polysciences, Hirschberg an der Bergstraße, Germany) on a glass slide and imaged using a Leica, TCS SP8X confocal microscope. Cell counting was normalized to the body surface of each specimen, as determined by ImageJ2 (Schindelin et al., 2015).

### Terminal deoxynucleotidyl transferase dUTP nick end labeling (TUNEL) assay

Planarians were fixed and stained for (TUNEL) as previously described (Almuedo-Castillo et al., 2014), with some modifications. After permeabilization, specimens were treated with 20 µg/ml proteinase K in PBST at 37°C. Samples were then bleached, incubated with terminal deoxynucleotidyl transferase (ApopTag Red *In Situ* Apoptosis Detection Kit, Millipore, Merck Chemicals) and stained with DIG-Rhodamine. Fluorescent images were acquired with a Zeiss LSM510 META (Carl Zeiss, Jena, Germany) mounted on an Axiovert 200M. Fluorescent cells were counted with the ImageJ (Schneider et al., 2012) ITCN (Image-based Tool for Counting Nuclei) plugin and their number was normalized to the body size of the animals, as determined by ImageJ.

### Fluorescent activated cell sorting (FACS)

Planarian dissociation and flow cytometry were performed as previously described (Moritz et al., 2012). Six fragments were pooled per sample, and three independent samples were measured for each experimental condition. Cells were stained with Hoechst 33342 (5 µg/ml; Thermo Fisher Scientific) and scored on a Gallios flow cytometer (Beckman Coulter, Krefeld, Germany).

### Real-time qPCR and data analysis

For qPCR, animals were snap-frozen in liquid nitrogen. After lysis in 200 μl RLT buffer (Qiagen, Venlo, The Netherlands) including 1% β-mercaptoethanol, RNA was isolated using phenol-chloroform, as previously described (Chomczynski and Sacchi, 2006); genomic DNA was removed with a Turbo DNA-Free Kit (Ambion, Thermo Fisher Scientific). SuperScript III First-Strand Synthesis SuperMix for qRT-PCR (Life Technologies) was used to reverse transcribe 250 ng RNA, following the manufacturer's instructions. After 1:10 dilution, complementary DNA was used as a template for qPCR, either on an ABI 7500 or an ABI 7900HT instrument (Applied Biosystems, Darmstadt, Germany), as previously described (Fernandez-Taboada et al., 2010; Pieters et al., 2016). Data generated were analyzed with the DDCt method using *Gapdh* as a reference gene and H_2_O- or GFP-injected animals as a reference sample.

### Carboxy HDFCA staining of ROS

An Image-iT LIVE Green Reactive Oxygen Species Detection Kit (Molecular Probes, Life Technologies) was used to visualize the production of ROS *in vivo*. Either Smed-p53(RNAi) or GFP(RNAi) animals were exposed to 50 µM CdCl_2_ or culture medium for 3 h prior to staining. Then, planarians were incubated in carboxy-H_2_DCFDA (25 µM, 1 ml), dissolved in their exposure solution, for 1 h prior to amputation. Amputated animals were again exposed to carboxy-H_2_DCFDA for 1 h before immobilization using 0.03% MS222 (ethyl 3-aminobenzoate methanesulfonate, Sigma-Aldrich, Taufkirchen, Germany) and 1% low melting point agarose (Life Technologies). Confocal imaging was performed for 75-120 min after amputation using a Zeiss LSM510 META mounted on an Axiovert 200 M (Carl Zeiss, Jena, Germany).

### 2D-DIGE

For each of the four experimental conditions examined, *S. mediterranea* (ten worms/sample with mucus layer removed) were shaken for 10 min at 70°C in lysis buffer [40 mM Tris HCl, 60 mM dithiothreitol (DTT), 2% sodium dodecyl sulfate (SDS) pH 8.5]. Upon removal of cellular debris, proteins were precipitated overnight in ice-cold trichloroacetic acid/acetone (20/80 v/v containing 0.1% DTT). Proteins were collected by centrifugation and solubilized in labeling buffer (7 M urea, 2 M thiourea, 4% Chaps, 30 mM Tris pH 8.5). Protein concentrations of the four thus obtained extracts were quantified using a 2D-DIGE Quant Kit (GE Healthcare, Amersham, UK). Aliquots from each extract were labeled with CyDye fluors (GE Healthcare) following the manufacturer's instructions. For intergel comparisons, an aliquot of Cy2-labeled internal standard (15 µg), prepared by pooling equal amounts of protein from each extract, was added to each gel sample. Gel samples were subjected to isoelectric focusing (Immobiline DryStrip, pH 4-7, 24 cm, GE Healthcare) followed by SDS-polyacrylamide gel electrophoresis (EttanDALTsix, GE Healthcare; 12.5% acrylamide gel). Gels were scanned with a Typhoon Variable Mode Imager (GE Healthcare) and resulting images were matched and analyzed with DeCyder 2-D Differential Analysis Software. Spots with fold changes in protein spot intensity ≥±1.5 were automatically picked with ProPic II using ProPic DIGE software (Digilab, Frankfurt/Main, Germany) and digested in gel using trypsin (Shevchenko et al., 2006).

### LC-MS/MS and data analysis

Tryptic digests were analyzed by LC-MS/MS as previously described (Jorissen et al., 2017), using an easy-nanoLC 1000 liquid chromatograph (Thermo Fisher Scientific) on-line coupled to a LTQ-Orbitrap Velos Pro (Thermo Fisher Scientific). Analysis of the mass spectrometric raw data was carried out using Proteome Discoverer software v.1.2 (Thermo Fisher Scientific) with built-in Sequest v.1.3 and interfaced with an in-house Mascot v.2.4 server (Matrix Science). MS/MS spectra were searched against dd_Djap_V1.contigs.orf.fasta and smedV4.contigs.orf_1.fasta databases (kindly provided by Jochen Rink, Max Planck Institute for Molecular Cell Biology and Genetics, Dresde, Germany). Search engine result files were evaluated in Scaffold v.3.6.1 (Proteome Software, Portland, OR, USA) using the Peptide Prophet and Protein Prophet algorithm, with a preset minimal peptide and protein identification probability of 95% and 99%, respectively.

### Statistical analysis

Cell counting and gene expression data were statistically tested for differences via two-way ANOVA using R (R i386 3.1.0). Subsequent multiple comparison testing was performed based on Tukey post hoc test, to detect differences between subgroups. Normality was tested according to the Cramer–von Miss and Anderson–Darlin normality tests. Homoscedasticity was evaluated based on graphical scatter plots and Bartlett test. If assumptions for normality or homoscedasticity were not met, a transformation of the data set was applied (either log or square root).

## Supplementary Material

Supplementary information
