# Supplementary material for: A carcinogenic trigger to study the function of tumor suppressor genes in Schmidtea mediterranea
Source: Dis Model Mech. 2018 Aug 16;11(9):dmm032573. doi: 10.1242/dmm.032573 (PMC6176991; doi:10.1242/dmm.032573)
Supplement: Supplementary information [file dmm-11-032573-s1.pdf]

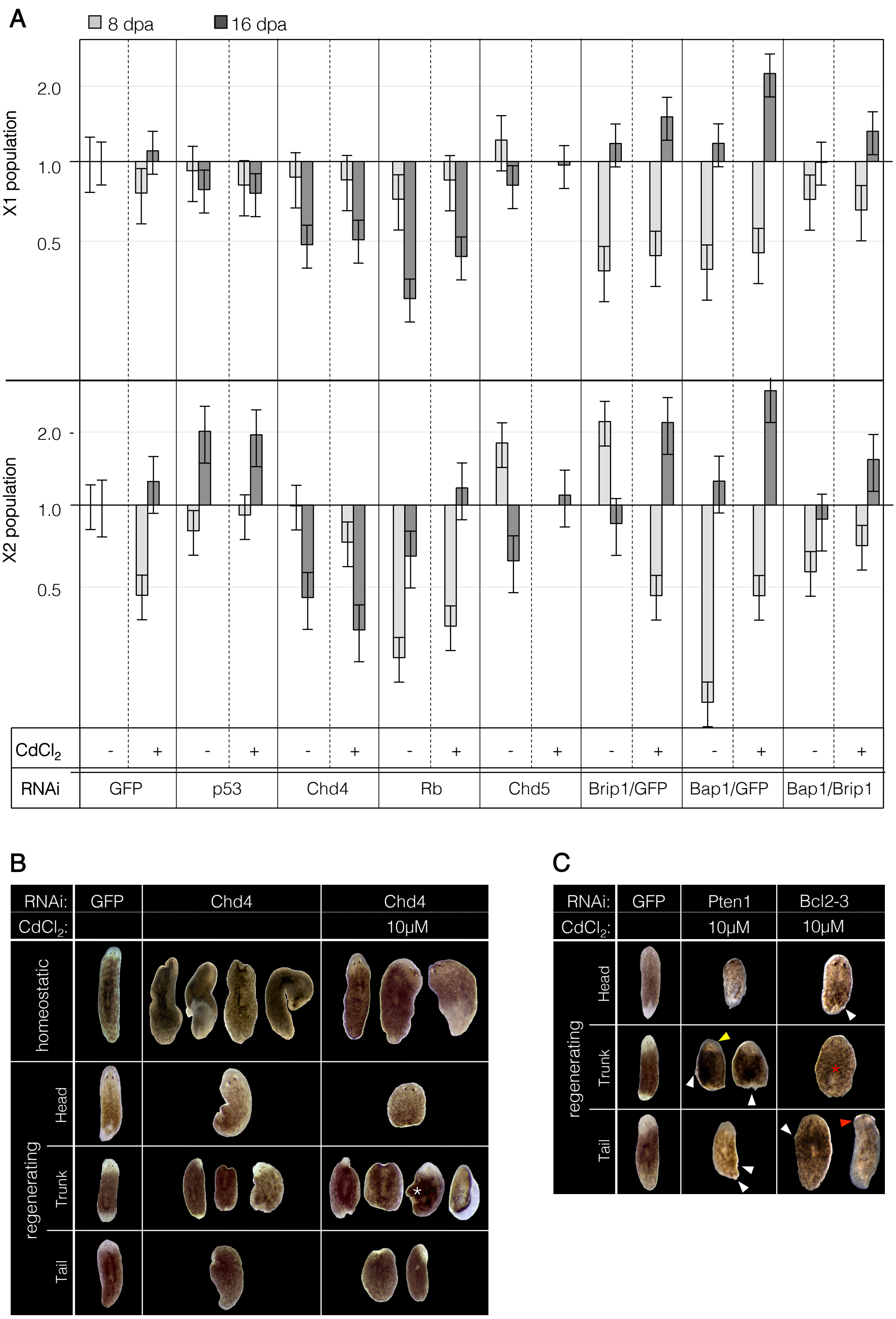

Supplemental figure 1. Phenotypes associated to TSGs is exacerbated by Cd. Changes in the stem cell populations (X1 and X2) associated with TSG KD, in presence (Cd-) or absence (Cd+) of Cd (A). The onset of Chd4(RNAi) phenotype is accelerated by Cd (B). Homeostatic animals imaged at 18 (Cd-) or 15 (Cd+) dpi; regenerating animals imaged at 15 (Cd-) or 11 (Cd+) dpa. Outgrowths marked with a white asterisk. Formation of small epidermal blisters (white arrowheads) and regeneration defects (cyclopia, yellow arrowhead) in Smed-Pten1(RNAi) (Cd+) fragments, at 12 dpa (C). Bloating (red asterisk), epidermal blisters (white arrowheads) and regeneration defects (lack of photoreceptor; red arrowhead) are visible in Smed-Bcl2-3(RNAi) (Cd+) animals between 11 and 14 dpa (C).

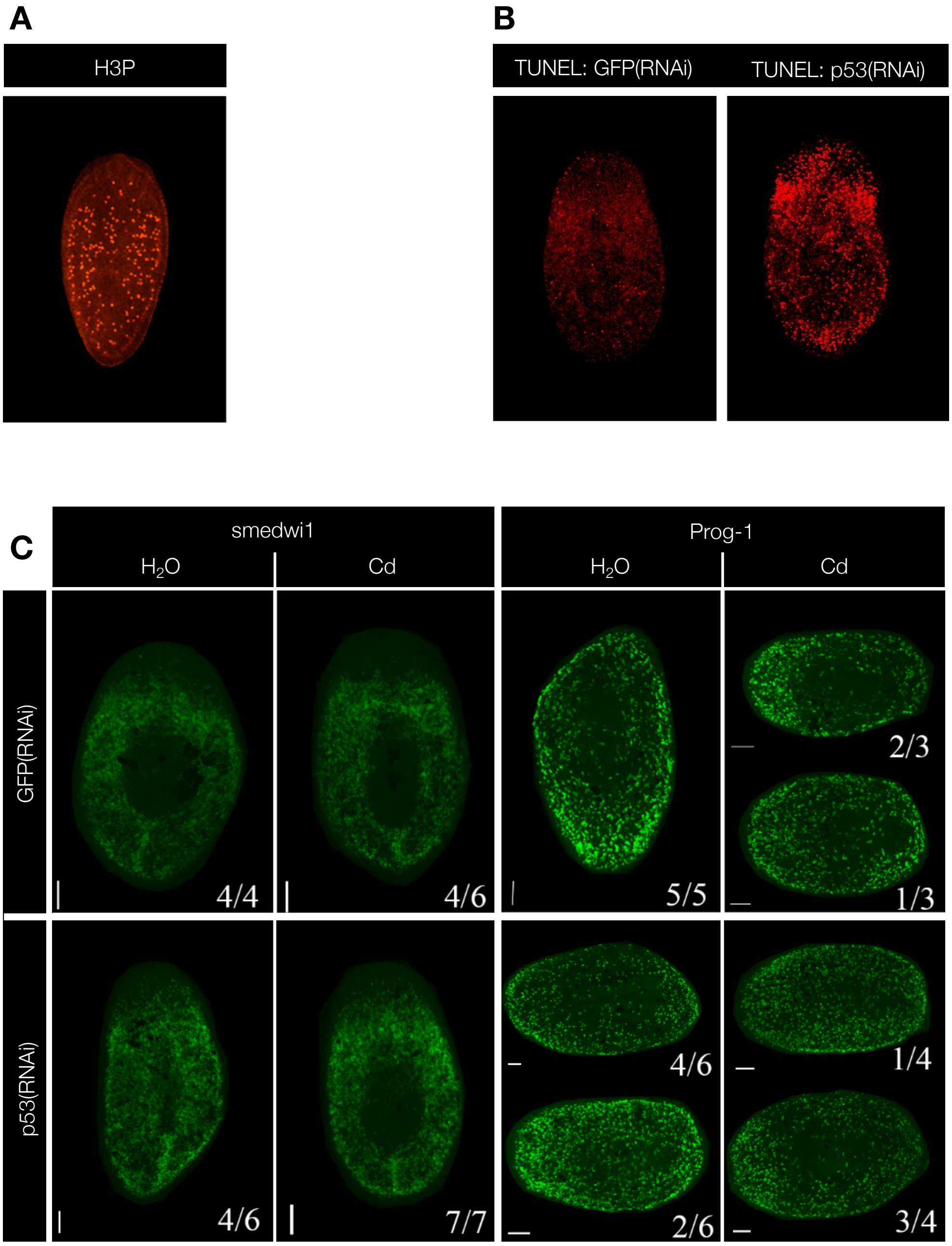

Supplemental Figure 2. Proliferation, apoptosis, stem cells and progeny in p53(RNAi) animals. Representative H3P immunostaining (**A**). Representative images of TUNEL in GFP(RNAi) (left) and p53(RNAi) (right), both exposed to Cd (**B**). Distribution of stem and progeny cells (**C**). smedwi1+ (C, left panels) and Prog-1+ (C, right panels) cells in GFP(RNAi) or p53(RNAi) animals at 11 dpa, in absence or presence of 10 μM $CdCl_2$. One confocal slice is shown for smedwi1; The maximum projection of all confocal slices are shown for Prog-1. Scale bars in C: 100 μM. Number of biological replicates is indicated in C.

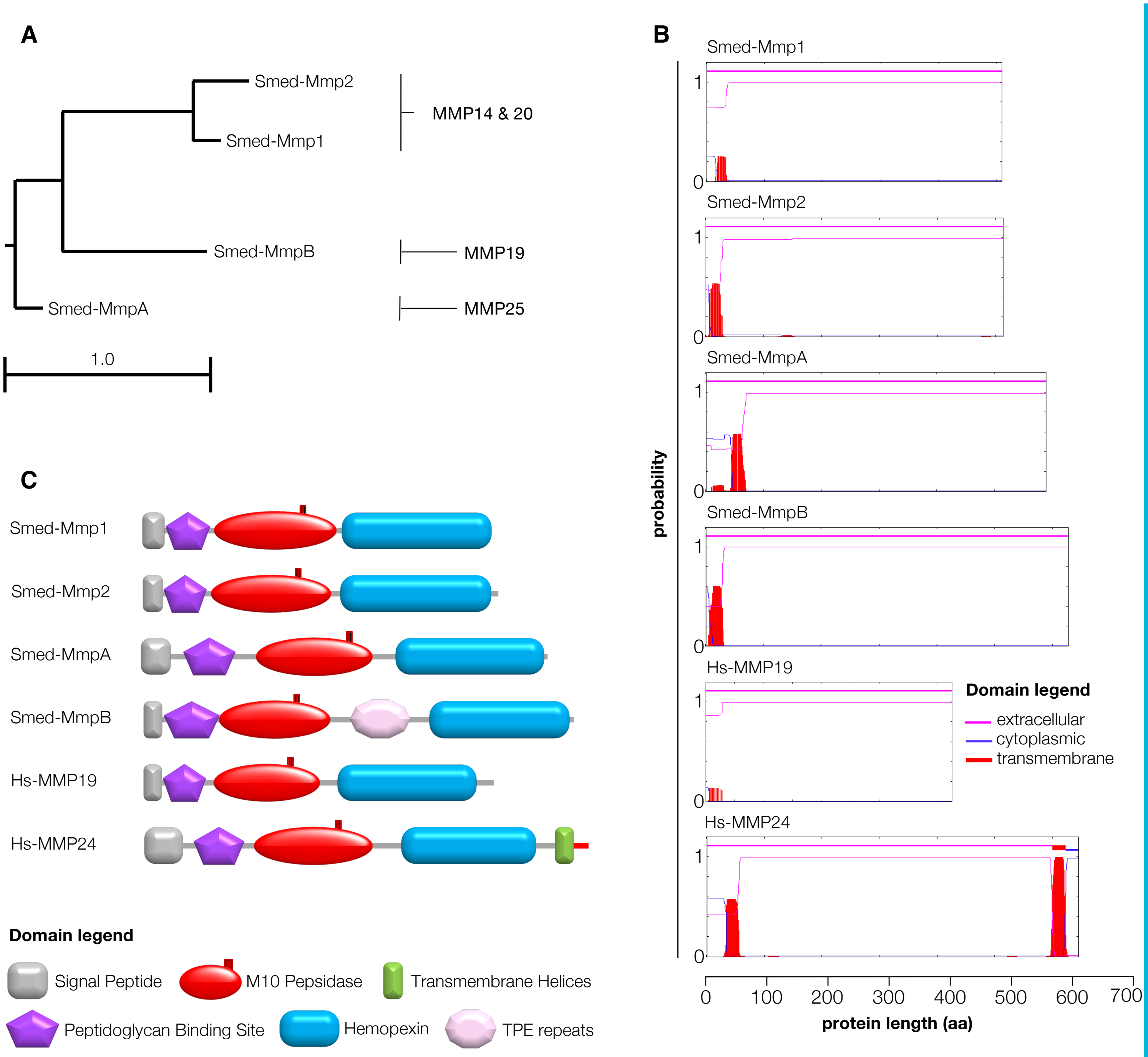

Supplemental figure 3. *In silico* predicted features of *S. mediterranea* MMP proteins. Genetic relationship of known planarian MMPs as depicted by Clustal Omega, and their homology with mammalian MMPs (A). Cytoplasmic, transmembrane and extracellular domains of planarian MMPs as predicted by TMHMM (http://www.cbs.dtu.dk/services/TMHMM/) compared with 2 human proteins, MMP19 (secreted) and MMP24 (membrane-bound) (B). Only Hs_MMP24 was predicted to have transmembrane and cytoplasmic domains. Protein domains of planarian MMPs, human MMP19 and human MMP24 as defined by InterProScan (https://www.ebi.ac.uk/interpro/) (C). Except for a stretch of TPE repeats, Smed-MMPB has the classical features of a secreted MMP: signal peptide in the N-terminus (SP), peptidoglycan binding domain (PGB), the proteolytic M10 domain and the hemopexin-like domain at the C-terminus. To be noticed the transmembrane helices at the C-terminus of the membrane-bound human MMP24.

Disease Models & Mechanisms • Supplementary information

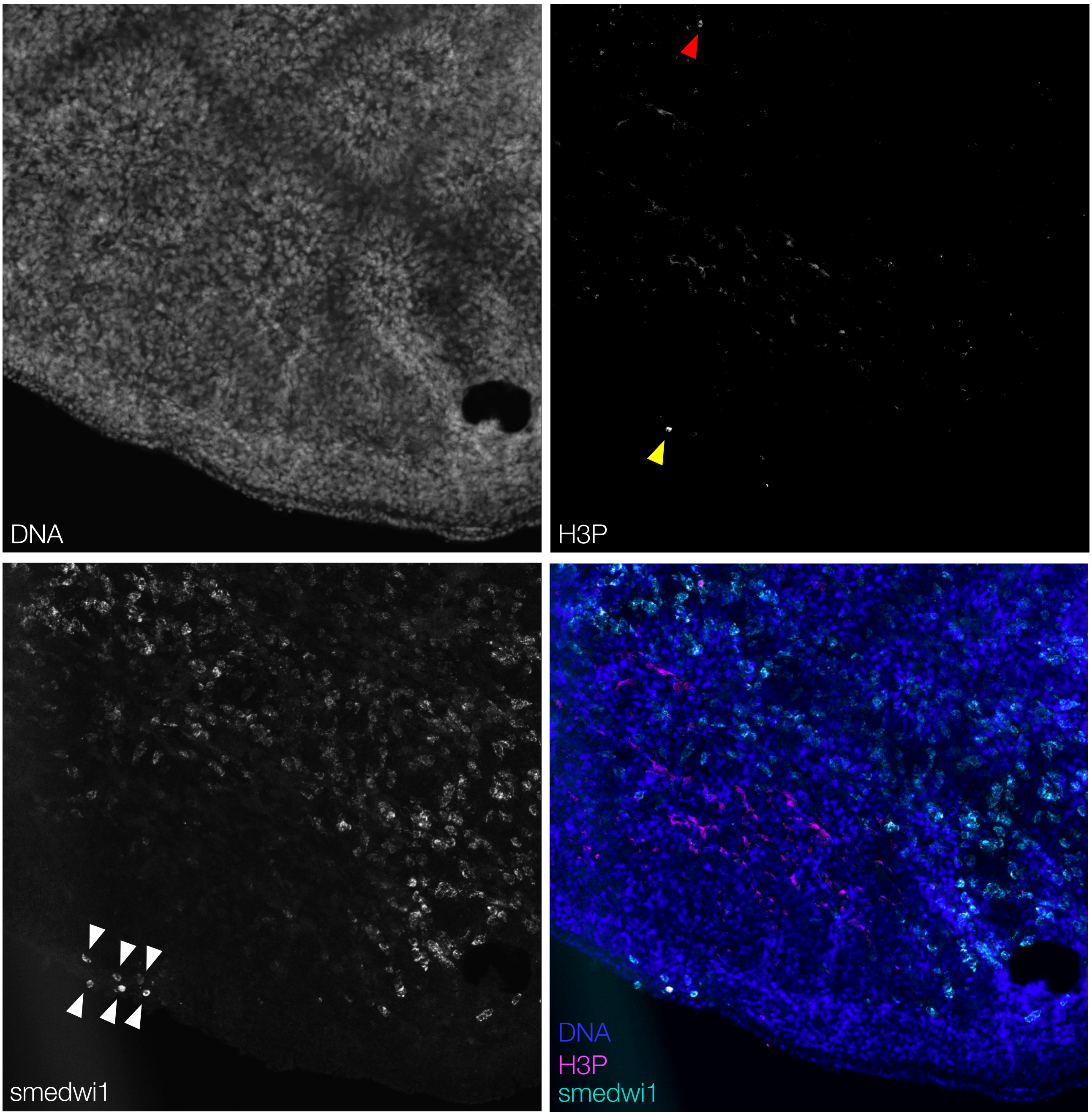

Supplemental figure 4. Confocal stack depicting a small epidermal blister in a Smed-MmpB(RNAi) animal. The blister was found at the lateral edge of the animal, posterior to the brain. It consisted of 6 smedwi1+ cells (white arrowheads), one of which was also H3P+ (yellow arrowhead). Within the depicted field, one additional H3P+ cell showed up in the body of the animal (red arrowhead).

Supplemental Table 1. Phenotypic changes after RNAi of the in silico defined TSGs in *S. mediterranea.*

Gene	Molecular function	Mutation - associated phenotype in human	Planarian ortholog expression	RNAi phenotype (homeostasis & regeneration)		Notes
				0 µM Cd	10 µM Cd	
p53	Transcription factor	Brain, breast cancer; leukemia	Stem and progeny cells (Pearson & Sánchez-Alvarado, 2010)	**Homeostasis**: head regression, thinning of pre-pharyngeal region (34/36, **94.4%**). **Regeneration**: abnormal blastema formation, impaired regeneration, especially of the head (H: 7/9 (**77.8%**), Tr: 5/9 **(55.5%**), Ta = 5/9 (**55.5%**).	**General**: faster onset of the phenotype, higher lethality. **Homeostasis**: ventral curling, head regression, thinning of pre-pharyngeal region, outgrowth formation (35/36, **97.2%**). **Regeneration**: abnormal blastema formation, impaired regeneration, outgrowth formation (H: 8/13, **61.5%**; Tr: 8/13, **61.5%;** Ta = 7/13, **53.8%**); increase of the X2 gate (188% @ 8 dpa, 183% @ 16 dpa).	
Rb	Transcriptional regulator	Retinoblastoma, osteosarcoma	Stem cells, post-mitotic cells (Zhu & Pearson, 2013)	**Homeostasis**: symmetrical lateral constrictions, head regression (34/36, **94.4%**). **Regeneration**: poor regeneration of all fragments (H: 14/21 (**67%**), Tr: 19/21 **(90.5%)**, Ta = 21/21 (**100%**); reduction of X1 (20% @ 11 dpa, 31% @ 16 dpa) and X2 (28% @ 11 dpa, 69% @ 16 dpa) gates.	**General**: stronger phenotype. **Homeostasis**: ventral curling, abnormalities of the pharynx and tail lesions (35/36, **97.2%**). **Regeneration**: abnormal blastema formation, little or no regeneration (H: 20/22, **91%**, Tr: 21/22, **95.5%**; Ta = 22/22, **100%**), outgrowth formation (H: 1/22, **4.7%**;Tr: 2/22, **9.4%**); reduction of X1 (27% @ 11 dpa, 45% @ 16 dpa) and X2 (19%@ 11 dpa) gates. At 16 dpa, X2 gate increases to 116%.	
Pten1	Transcriptional regulator	Breast, thyroid, head and neck cancer; glioma	Progeny cells (Oviedo et al., 2008)	**Regeneration**: regeneration impaired; pigmented dots; death (4/6, **66.7%**).	**General**: faster onset of the phenotype **Regeneration**: regeneration impaired; pigmented dots (3/12, **25%**); blisters (4/12, **33%**), bloating (1/12, **8.3%**), and death (5/12, **41.6%**).	
Chd4	Helicase (transcriptional repressor)	Hyper-methylation in nasopharyngeal carcinoma	Stem and progeny cells (Scimone et al., 2010)	**Homeostasis**: asymmetrical lateral dents, head regression (34/36, **94.4%**). **Regeneration**: regeneration impaired (50/54, **92.5%**); reduction of X1 (49% @ 16 dpa) and X2 (45% @ 16 dpa) gates.	**General**: faster onset of the phenotype. **Homeostasis**: asymmetrical lateral dents, head regression (34/36, **94.4%**). **Regeneration**: regeneration impaired (53/54, **98.1%**), outgrowth formation (H: 1/18, **5.5%**); X1 (85% @ 11 dpa, 50% @ 16 dpa) and X2 (72% @ 8 dpa, 34% @ 16 dpa) gates were further reduced.	
Chd5	Helicase (transcriptional repressor)	Epithelial, neural and hematopoietic malignancies	Stem cells, post-mitotic cells	**Regeneration:** increase of both X1 and X2 gates, limited to the early phase (8-10 dpa: X1 120%; X2 170%), then reduction of both gates (15-17 dpa: X1 81%; X2 61%).	N/A	
Wt1	Transcription factor	Kidney, ovarian cancer; leukemia	Post-mitotic cells	N/A	N/A	
Bap1	Transcriptional repressor	Breast, lung, skin, kidney cancer; metastasis	Post-mitotic cells	**Regeneration**: reduction of both X1 and X2 gates, limited to the early phase (8-10 dpa: X1 40%; X2 18%).	**Regeneration**: reduction of both X1 and X2 gates, limited to the early phase (8-10 dpa: X1 40%; X2 46%), then increase of both gates (15-17 dpa: X1 210%; X2 267%)	No phenotype in Brip1/Bap1 double RNAi

Gene	Molecular function	Mutation - associated phenotype in human	Planarian ortholog expression	RNAi phenotype (homeostasis & regeneration)		Notes
				0 µM Cd	10 µM Cd	
Brip1	dsDNA repair	Breast, ovarian, germline cancer	Stem cells, post-mitotic cells	**Regeneration**: reduction of X1 gate and increase of the X2 gate, limited to the early phase (8-10 dpa: X1 39%, X2 205%).	**Regeneration**: reduction of both X1 and X2 gates, limited to the early phase (8-10 dpa: X1 45%, X2 205%); late increase of both X1 and X2 populations (15-17 dpa: X1 146%, X2 203%)	No phenotype in Brip1/Bap1 double RNAi
Bcl2-3	Pro-apoptotic regulator	Colonrectal cancer, Leukemia	N/A	**Regeneration**: 1 head fragment died (1/9, **11.1%**)	**Regeneration**: regeneration impairment (7/9, **77.8%**), epidermal blisters (1/9, **11.1%**), bloating (2/9, **22.2%**), outgrowth formation (1/9, **11.1%**) and death (5/9, **55.5%**); impaired movement (7/9, **77.8%**)	Pigmentation change (pigment clusters)
Msh2	DNA mismatch repair	Colorectal cancer	Stem cells (Hollenbach et al., 2010)	N/A	N/A	
Mlh1	DNA mismatch repair	Stomach, head and neck, colorectal, lung cancer	Stem cells	N/A	N/A	
Wwox	protein–protein interaction	Head and neck, uterus, stomach cancer	Post-mitotic cells	N/A	N/A	
Pdcd4	Cell cycle and transcriptional regulator	Lung, liver, colorectal, breast cancer; glioblastoma	Post-mitotic cells	N/A	N/A	
Max	Transcriptional regulator	Lung cancer	Stem cells, post-mitotic cells	N/A	N/A	
Apc	Signaling	Colorectal cancer	Post-mitotic cells	N/A	N/A	
Smg1	Kinase (NMD)	Leukemia	Stem cells, post-mitotic cells (Gonzalez-Estevez et al., 2012)	N/A	N/A	
Mta1	Transcriptional coregulator	EMT, invasion, metastasis	Stem cells	N/A	N/A	
Lrh1	Nuclear receptor and transcription factor	Pancreatic cancer; cell proliferation	Post-mitotic cells	N/A	N/A	

Supplemental Table 2. qRT-PCR oligonucleotides used in this study.

Gene Name	Sequence 5' → 3'
Smed-Gapdh	P. forward GAGTTGGAATCAATGGCTTCG<br>Probe CGCGCAACACCAATCGTCCAATTC<br>P. reverse TCAACTGTGCCTTTCTCCAG
Smed-piwi-1	P. forward AGTTCCTGTTCCAACGCATTATG<br>Probe CTGAACTCGTTGGCAAGA<br>P. reverse CTGGAGGAGTAACACCACGATGA
Smed-pcna	P. forward GTGATGGTTTTGAGACTTATCGATG<br>Probe TGTTAGGGAATCATTACTACCAAGCGCC<br>P. reverse GTTTCACTTGAATCAGCGGC
Smed-inx13	P. forward TTCTGTTTCTCAGGTCGATTTCT<br>Probe TCAAACAATCGGCAAACAACGCTCG<br>P. reverse CCATGAACGTTGGCGATTTG
Smed-smad6/7	P. forward GCCACAGTGAGTCAGGTTTA<br>Probe ACCAGTCATGCCCATCTATCACGAC<br>P. reverse CACCAGCGATTTCCAGTTTG
Smed-soxP-1	P. forward TCAACACCACTAAGCACCTATC<br>Probe CACACGTAAGCTGAGAACGCCTGA<br>P. reverse CAGCTGCAATTTGGCCTATG
Smed-soxP-2	P. forward GACTTTAACCATGAGCCGATTG<br>Probe CAACCGATTCCAGTTCAACGATTGCC<br>P. reverse CCCGTTCCATCTATCAGAAACT
Smed-egr-1	P. forward TCGGACAATTCGAACAGGTAAA<br>Probe CGGGTGGCAGTTGATTGGATTTGC<br>P. reverse CGATCAGTACAATTTCGAGAGAGG
Smed-fgfr-1	P. forward CTCCAGACGCTAGTTCCATTATAG<br>Probe CGATGGCGACCGATTTGTTGCAT<br>P. reverse GGACAAGACATGCTGTTTGATG
Smed-soxP-3	P. forward GAAGCTGCTTGGCCTCATTA<br>Probe CGGAGTCCGTTCTTCAGCTGACATT<br>P. reverse GGCTAGCCAATATCCGAATTTCT
Smed-zfp-1	P. forward TCCCGTGCCTGAACAATTT<br>Probe TGTCACATTTGCAACACCAGCTTCAC<br>P. reverse CGCATGCCTCTGTAGATTTGA
Smed-p53	P. forward ATCGTCGAGCCTGTTTCATC<br>Probe TCCGACGACATGCCAACATTGTCT<br>P. reverse ATCAAATTCTCCGTTGGGAATAAAG
Smed-gata4/5/6	P. forward GTGAACTGTGGAGCTAGCAATA<br>Probe TTGTGGTCCCGGGATAATTCTGGC<br>P. reverse AGAGAACCTGTCGCATTCATC

Smed-hnf-4	P. forward TTTGGAAGCGACTTGGTATAGG<br>Probe TGTCGTTGATCCGTCGCTTCTTGT<br>P. reverse CTAATCCACCCAGCTCTTTCTG
Smed-nkx2.2	P. forward CCGATTTCAAACAGTTCCACTTAC<br>Probe TGCCAGCAGACTCAAACATCCAGT<br>P. reverse CAGTGATCCGTACGCTGAATTA
Smed-prox-1	P. forward GATAAAGTCAGCCGGAATAGCA<br>Probe ACGTCCTCAATGTGCTGTAAAGTGCA<br>P. reverse CGCCTTCTTGATTTAGCAAAGAC
Smed-agat-1	P. forward GGTTGGAAGATTGTGAAGGG<br>Probe TGTATGAAGGCATGAGTTACAAGTGGC<br>P. reverse CCAACCTCTCGCTTTTCA
Smed-NB32.1g	P. forward GGCACTCATTTCTCGTTTCTGTATT<br>Probe TGTCGAGTCGCATTTTAAATCGGCG<br>P. reverse GTTCTCGCTGTGTTATTTGTTTACGT
Smed-msh-2	P. forward GTGCCTTTGCGACTCATTTC<br>Probe ACACGTGACTGCACAGACAATTGGA<br>P. reverse GGCCCTTCCTCGACTTTATAC
Smed-mta-1	P. forward TCCGTGACAGCCCATTTATATC<br>Probe TCCGACAAGCTGTTCAACTCCACA<br>P. reverse CAACAAGTTCCATAGAGTCCAATAAC
Smed-mlh1	P. forward ATTAGCGAGTGTTACCCATGTAG<br>Probe CTGTTGAAACCACCCAAACCGTGT<br>P. reverse TCGAACGATTGTTCCGGTATT
Smed-Rb	P. forward GCAGTTTGCGACTGAGAGATA<br>Probe CGATCGATTGCACATCAGCCGAGA<br>P. reverse CATCGGTCCTTGAGAAGATGAG
Smed-smg-1	P. forward CCTCCTGGTTCATGGACTAATG<br>Probe TGTTTGCTCGGTTGAAGCACAGTG<br>P. reverse GTTGCTGGAAATCGCCAATC
Smed-brip-1	P. forward CCCGTCAAACATCAGAAATGAAT<br>Probe AGATGGCGCATTATTGCTAGCGGT<br>P. reverse CTACACCTTCACTGGCCTTAC
Smed-Chd5	P. forward TTGAGAACGCGTTGCTTATTG<br>Probe TGGGAGCTGCATGTGACGGATATT<br>P. reverse GGGACGGATTGATGTGGATAG
Smed-pten-1	P. forward CGTTTGTGCTTGTTTGCTATCT<br>Probe ATCCGCCACGGACATTCTCCAATT<br>P. reverse TTGTCACTCCTTTCCCGTTC
Smed-pdcd-4	P. forward AGCGCCAGAAATAGTTGGTAAA<br>Probe AGCCGTAGCCGACGATTTATTAGCC<br>P. reverse GCTAAAGCGCGGAGTTGATA

Smed-wt-1	P. forward CGATGAGCTGTCTCGACATAAA<br>Probe AGTTCAATCGGAGCGATCATTTGTCCA<br>P. reverse TGGAATGATTCTTGTGGGTCTT
Smed-wwox	P. forward GAATGGAGGTCTGAGCGTAAT<br>Probe AGTGCCGTAAAGCCTTGCAAATGC<br>P. reverse CGCTATCGGTCTGAAAGTTAGT
Smed-bap-1	P. forward CGAGGATTAGCACTGGGAAAT<br>Probe CAGAATTGGCGGATGCACACAACA<br>P. reverse GCGGCAACACTGTGATATAAAC
Smed-Apc	P. forward GGAAGGGACACCGAATAGTTT<br>Probe TCGTTTCAGAGCAGATGGAATCACGC<br>P. reverse AGCACCTGGTGGTTTAGAATAG
